# Extracellular Vesicles From Glioblastoma Cells Reflect 2D vs. 3D Culture Adaptation and Resistance to Temozolomide

**DOI:** 10.1016/j.mcpro.2026.101610

**Published:** 2026-06-24

**Authors:** Getulio Pereira de Oliveira, Alan Zimmerman, Cintia C. Palu, Vivian Tran, Gabrielle Bogut, Stephanie Chidester, John Tigges, Rafael A. Vega, Elena Aikawa, Jennifer Jones, Ionita C. Ghiran, Alexander R. Ivanov

**Affiliations:** 1 Department of Chemistry and Chemical Biology, Barnett Institute of Chemical & Biological Analysis, Northeastern University, Boston, Massachusetts, USA; 2 Graduate Program of Genomic Sciences and Biotechnology, Catholic University of Brasilia, Brasilia, Brazil; 3 Expert Biomarker Data Scientist, Sanofi, Cambridge, UK; 4 Department of Anesthesia, Beth Israel Deaconess Medical Center, Harvard Medical School, Boston, Massachusetts, USA; 5 Center for Interdisciplinary Cardiovascular Sciences, Brigham and Women’s Hospital, Harvard Medical School, Boston, Massachusetts, USA; 6 Laboratory of Pathology, Center for Cancer Research, National Cancer Institute, Bethesda, Maryland, USA; 7 Nanoflow Cytometry Core Facility, Beth Israel Deaconess Medical Center, Harvard Medical School, Boston, Massachusetts, USA; 8 Division of Neurosurgery, Beth Israel Deaconess Medical Center, Harvard Medical School, Boston, Massachusetts, USA

**Keywords:** extracellular vesicles, glioblastoma, spheroid, U87 cells, temozolomide

## Abstract

Glioblastoma (GBM) is an aggressive brain tumor marked by extensive heterogeneity, resistance to therapy, and dismal prognosis. Extracellular vesicles (EVs) have emerged as key players in GBM biology, mediating intercellular communication and therapy adaptation. However, the exact functions and molecular impact of EVs in GBM remain incompletely understood. In this study, we performed a comparative proteomic analysis of U87MG GBM cells grown in two-dimensional (2D) monolayers and three-dimensional (3D) spheroids following temozolomide (TMZ) treatment, alongside characterization of EVs derived from both culture systems. 3D-spheroids secreted more EVs of smaller size and exhibited a more TMZ-resistant, stem-like proteome under TMZ-induced genotoxic stress. In contrast, 2D cell cultures demonstrated greater proteome remodeling, with EVs enriched in protein families involved in DNA repair, oxidative stress adaptation, and methylation processes. Notably, several methyltransferases were decreased intracellularly but selectively retained in EVs, suggesting active sorting to influence the tumor microenvironment or modulate epigenetic states in recipient cells. EVs also carried adhesion molecules and signaling proteins linked to migration, invasion, and Wnt pathway activation, as well as metabolic enzymes connecting serine metabolism and redox control to TMZ resistance. Mapping EV and cellular proteomes onto The Cancer Genome Atlas (TCGA) dataset identified prognostic protein families associated with either poor or favorable patient outcomes. Our data demonstrate that EV cargo composition mirrors TMZ-induced phenotypic adaptation and reveals molecular mechanisms underlying therapeutic resistance. These EV-associated signatures may serve as clinically actionable biomarkers for patient stratification and offer potential targets to overcome chemoresistance in GBM.

## Highlights

• 3D spheroids reshape glioblastoma response to temozolomide treatment.
• EV proteomes differ between 2D and 3D glioblastoma culture models.
• 3D models show reduced proteomic remodeling under TMZ exposure conditions.
• EV cargo reflects metabolic and stress adaptation pathways in GBM cells.
• Tumor architecture impacts EV signatures and drug response dynamics.

## Graphical Abstract

**Figure:**
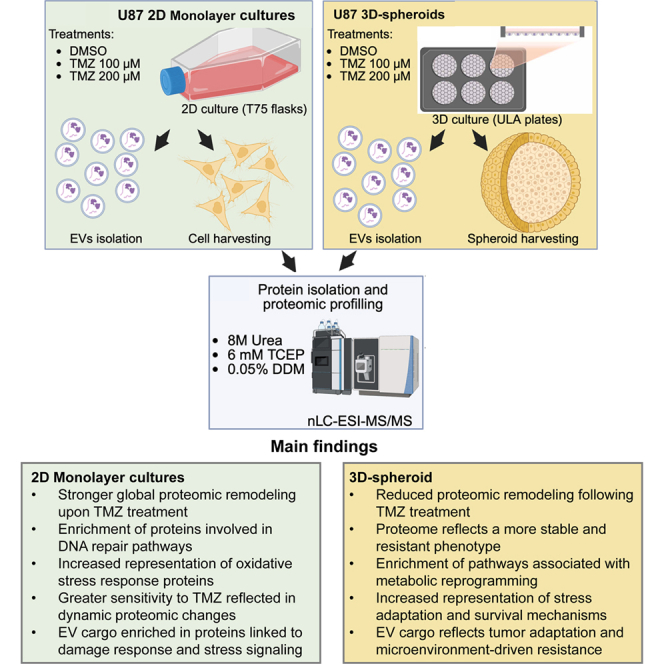

## In Brief

Proteomic profiling of the U87MG glioblastoma cell line and extracellular vesicles (EVs) reveals that 3D spheroid architecture reshapes cellular responses to temozolomide and alters EV cargo composition. Compared to 2D cultures, 3D models exhibit reduced proteomic remodeling and distinct EV signatures linked to stress adaptation and metabolic reprogramming pathways. These findings highlight the importance of tumor architecture in drug response and support applications of EVs as potential indicators of glioblastoma state, progression, heterogeneity, and therapeutic response in clinically relevant contexts.

Glioblastoma (GBM) is the most aggressive (WHO grade IV) malignant primary brain tumor, representing 15% of all brain tumors. It has a very poor prognosis for survival, with a median survival of less than 2 years (1), 1-year relative survival of 41.4%, and 5-year relative survival of 5.4% (2). Magnetic resonance imaging (MRI) is the primary diagnostic tool for GBM, and currently, there are no specific blood biomarkers known for GBM. Non-invasive approaches capable of detecting molecular changes in real time are critically needed to overcome the limitations of imaging and tissue sampling in GBM management. When a tumor biopsy is performed, some genetic markers, such as IDH mutations, 1p19q deletion, MGMT promoter methylation, and EGFRvIII amplification, are frequently tested in routine clinical practice implemented during the last decade (3). The treatment is impaired by the poor ability of drugs to cross the blood-brain barrier, which prevents efficient passage of cancer therapeutics, including small molecules and antibodies (4). Radiotherapy (RT) with concomitant and adjuvant use of the chemotherapy alkylating agent temozolomide (TMZ) is the current standard of care for patients with newly diagnosed GBM up to age 70(5). TMZ is an oral alkylating agent that has become a cornerstone in the treatment of glioblastoma; it works by adding methyl groups to the DNA of rapidly dividing tumor cells, leading to DNA damage and cell death (6). The standard treatment protocol for GBM includes surgical resection, followed by radiotherapy and concomitant TMZ, then continuing with adjuvant TMZ therapy (7). However, the DNA repair protein O^6^-methylguanine-DNA methyltransferase (MGMT) present in GBM can cause resistance to TMZ, decreasing its efficacy (8). TMZ disrupts normal base pairing, leading to replication errors that activate the mismatch repair (MMR) system. Persistent MMR activity results in double-strand breaks and ultimately induces apoptosis. However, MGMT removes the methyl group from O^6^-MeG, restoring the DNA to its original state, thus preventing damage accumulation. As a result, GBM cells with high MGMT expression levels can efficiently repair TMZ-induced lesions, rendering the treatment ineffective (9).

Recently, extracellular vesicles (EVs) became promising candidates for biomarker discovery due to their natural ability to transport and deliver biomolecules (proteins, miRNAs, lipids, etc.) between different cells, tissues, and organs that recapitulate the pathophysiological status of the parental cell (10, 11). EVs are nanosized (50–200 nm) bilipid layer-enclosed vesicles, categorized as exosomes (biogenesis through the endosomal pathway) or microvesicles (biogenesis through the budding of the plasma membrane) (12). Additionally, other non-vesicular extracellular particles, such as exomeres and supermeres, have also been implicated in various biological processes (13, 14). In GBM, the ability of EVs to be sampled from biofluids has led to growing interest in their potential use.

Spheroid/organoid models have been used extensively to study a wide range of cancers, such as ovarian (15, 16), prostate (17), colorectal (18, 19), pancreatic (20), contributing to the understanding the role of EVs in disease evolution, response to treatments, and biomarker discovery. 3D models more accurately capture and reproduce disease-specific protein signatures, offering a superior representation of pathophysiology compared to traditional 2D systems (21). The utilization of U87 spheroid models and EVs derived from U87 cell lines represents a significant advancement in GBM research, bridging the gap between basic scientific exploration and clinical application (22). This cell line was isolated from malignant gliomas, and it is considered tumorigenic in nude mice. U87 spheroids, which mimic the 3D architecture and microenvironment of GBM tumors more accurately than traditional 2D cultures, provide an invaluable platform for studying tumor growth, invasion, and drug resistance mechanisms (23, 24). By capturing the complex interplay of tumor cells in a controlled setting, these models facilitate the identification of novel biomarkers and therapeutic targets (25). Moreover, the use of U87-derived EVs as a proxy for tumor-derived EVs present in patient blood samples can enhance the development of non-invasive diagnostic and monitoring tools. Such translational approaches not only accelerate the pace of glioblastoma research but also hold the promise of improving personalized treatment strategies, ultimately leading to better patient outcomes in this challenging malignancy (26).

U87 glioblastoma cells were selected for this study as a well-characterized and widely used experimental model that enables reproducible, large-scale proteomic profiling under controlled culture conditions. While U87 cells do not fully recapitulate the genetic and microenvironmental heterogeneity of primary glioblastoma, their robustness and scalability make them well-suited for systematic discovery-based analyses comparing two-dimensional and three-dimensional growth architectures and their associated extracellular vesicle profiles. In this study, we used high-resolution mass spectrometry (nLC-ESI-MS/MS) to comprehensively profile the proteomes of extracellular vesicles (EVs) derived from temozolomide-treated U87 glioblastoma cells cultured in two-dimensional (2D) and three-dimensional (3D) models, with the goal of identifying trends in molecular pathway changes and plausible protein biomarker candidates associated with therapy response, tumor heterogeneity, and glioblastoma progression.

## Experimental procedures

### Cells and Reagents

U87 MG cell line (referenced as U87 in other (i.e., non-experimental) sections of the manuscript for simplicity) was obtained from American Type Culture Collection (ATCC, cat# HTB-14). Dulbecco's phosphate-buffered saline (dPBS, cat# 14190250), Dulbecco's Modified Eagle Medium, high glucose (DMEM, cat# 11965092), Fetal Bovine Serum (FBS, cat# A5670701), Penicillin-Streptomycin (P/S, 10,000 U/ml, cat# 15140122), Dead Cell Apoptosis Kits with Annexin V for Flow Cytometry (cat# V13241), Hoechst 33342 (cat# H3570), CellMask Plasma Membrane Stains (deep red, cat# C10046) were obtained from Thermo Fisher Scientific. Temozolomide was obtained from Millipore Sigma (Burlington, MA, cat# T2577). For proteomics, Optima LC-MS grade water and acetonitrile (ACN) for LC-MS/MS experiments were purchased from Fisher Scientific. HPLC-MS grade formic acid (FA), urea, and iodoacetamide (IAA) were obtained from Sigma-Aldrich. Empore C18 solid-phase extraction disks were obtained from 3M. Lysyl endopeptidase (Lys-C) was obtained from Wako Chemicals. Ammonium bicarbonate (ABC) was purchased from Honeywell Fluka. Tris(2-carboxyethyl)phosphine (TCEP) was obtained from Thermo Fisher Scientific. Trypsin Gold was purchased from Promega.

### U87 MG 2D Culture

Cells were thawed, washed in full media (DMEM + 10% FBS + 1% P/S), and seeded in T25 or T75 EasYFlask (Thermo Fisher) until 90% confluence. Cells were cultured in full media at 37°C with 5% CO_2,_ and passages were performed every 3 days. For 2D and 3D experiments, U87 cells were cultured in three T-225 flasks containing full media DMEM +10% FBS +1% P/S (50 ml media in each flask) until they reached approximately 80% confluence (∼30 × 10^6^ cells). Cells were harvested, stained with Trypan blue, counted by a hemocytometer, and reseeded in Elplasia plates (three 6-well plates) or T-75 flasks (18 flasks) using the desired concentration of cells. We considered individual T-75 flasks as 2D replicate samples (6 DMSO treated, 6 TMZ 100 μM, and 6 TMZ 200 μM treated).

### U87 MG spheroid (3D) Culture

Cells cultured in T225 flasks were harvested, counted in the hemocytometer and 1000 cells per microcavity (2,88 × 10^6^ cells/well) were seeded into three 6-well ultra-low attachment (ULA) U-shaped plates (Corning Elplasia 6-well black/clear round bottom ULA microcavity plate, with Lid, cat# 4440) in 3 ml of full media (DMEM + 10% FBS + 1% P/S). For the formation of larger spheroids (approximately 1 mm in diameter), we used a 96-well ULA plate (Corning Elplasia 96-well black/clear round bottom ULA microcavity microplate, with lid), and 20,000 cells were seeded per well in 200 μl of full media. The formation of U87 spheroids (3D) cells was monitored daily by an inverted microscope. For 2D culture experiments, cells from T225 flasks were harvested, counted, and 2 × 10^6^ cells were seeded in 18 T-75 flasks. Cells cultured in 2D flasks and 3D plates were maintained in full media (DMEM + 10% FBS + 1% P/S) for 96 h at 37°C with 5% CO_2_. We considered individual wells of a 6-well Elplasia plate as 3D replicates (6 DMSO-treated, 6 TMZ 100 μM, and 6 TMZ 200 μM).

### Temozolomide Treatment

After 96 h, the 2D flasks and 3D plates were carefully washed 3 times with prewarmed PBS to guarantee the removal of FBS. After washing, 15 ml or 5 ml of pre-warmed DMEM (without FBS and P/S) was added to 2D Flasks and 3D plates for treatment, respectively. Temozolomide was dissolved in anhydrous DMSO prior to treatment and used at concentrations of 50, 100, and 200 μM. 2D and 3D cell cultures were maintained for 72 h under treatment at 37 °C with 5% CO_2_ and used for EV harvesting. After 72 h, 2D and 3D cell cultures were imaged, the cell culture media were collected for EVs isolation, and cells/spheroids were lysed for downstream analysis. Prior to EV collection, DMSO-treated cells exhibited 95% viability by trypan blue staining.

### Annexin-V-PI fluorescence assay

The effect of TMZ on U87 cell death was assessed by Annexin-V-PI assay, following the manufacturer’s instructions. Briefly, 1X annexin-binding buffer was prepared by diluting 1 ml of 5X annexin-binding buffer to 4 ml of deionized water. Propidium iodide (PI) solution was prepared by diluting 5 μl of the 1 mg/ml PI stock solution in 45 μl 1X Annexin-binding buffer. Cells cultured in 2D and 3D conditions were washed with PBS once, and 1X annexin-binding buffer was added to the cultures. Then, 5 μl Alexa Fluor 488 Annexin V and 1 μl 100 μg/ml PI working solution were added to each T25 Flask (2D model) or each ULA plate well (3D model). Nuclei were visualized by adding 1 μl of Hoechst 33342 to both cell conditions for 15 min. Cells were imaged using an Olympus IX73 inverted microscope (Olympus and the 10 × 0.45 PlanFluorite and 20 × 0.70 PlanFluorite objectives with DAPI, FITC, and Texas Red filter sets. Quantification of the fluorescence images was performed using ImageJ (National Institutes of Health (NIH)). Briefly, merged fluorescence images were split into individual 8 bit channels, inverted, the threshold was set, converted into binary, and the rectangle tool was used to measure the fluorescence intensity in the 2D cell culture images. For 3D models, the ellipses tool was used to measure the fluorescence intensity in the 3D-spheroids images. The Annexin V and PI fluorescence intensities were normalized by Hoechst 33342 intensities in each image.

### EV Isolation

After 72 h of temozolomide (TMZ) treatment, cell culture media from the 2D model (T-75 flasks, 15 ml) and the 3D model (ultra-low attachment plates, 5 ml per well) were collected for extracellular vesicle (EV) isolation. Serum-free conditioned media were subjected to sequential centrifugation at room temperature (300 × *g* for 5 min, 2000 × *g* for 10 min, and 4500 × *g* for 20 min), with the supernatant transferred to fresh tubes after each step to remove cells and cellular debris. Clarified supernatants were then concentrated to a final volume of 500 μl using Pierce 15 ml protein concentrators with a 100 kDa molecular weight cut-off (MWCO) polyethersulfone membrane (Thermo Fisher Scientific). This pre-SEC concentration step was performed to reduce sample volume and remove low-molecular-weight soluble proteins, thereby facilitating efficient chromatographic separation. Each concentrated sample (500 μl) was subsequently loaded onto a qEV original 35 nm Gen 2 size-exclusion chromatography (SEC) column (Izon Science). The void volume (first 2.5 ml, corresponding to the buffer volume) was discarded, and EV-enriched fractions were collected in the subsequent 1.8 ml according to the manufacturer’s instructions. Following SEC, EV-enriched fractions were reconcentrated to a final volume of 50 μl using Pierce 1.5 ml protein concentrators with a 100 kDa MWCO membrane to obtain sufficient material for downstream biophysical and proteomic analyses.

### Fluorescence Microscopy of EVs Using Lipophilic Dye

CellMask plasma membrane stains (ThermoFisher#C10046), a deep red dye, was prepared by diluting the stock 1:10 with 1X PBS. EV samples were prepared by pooling 1 μl of each EV replicate isolate (a total of 6 μl per condition). Then, 6 μl of EVs isolated from 2D and 3D conditions (treated or untreated) were incubated with 1 μl of the dye for 30 min. As negative controls, PBS (blank) and void samples underwent the same labeling steps as EV samples. After incubation, 5 μl of the samples were deposited on a glass slide with coverslip and visualized using a 100X objective of an inverted Olympus IX73 fluorescence microscope with a Texas Red filter set.

### Negative Staining Transmission Electron Microscopy of EVs

Carbon-coated grids were glow-discharged for 30 s to increase the overall hydrophilicity of the surface. A drop containing 5 μl of pooled EVs was placed on parafilm, and then a carbon-coated copper grid was placed on the top of the drop for 15 min. The grids were then washed once with ultrapure water (Milli-Q), stained with 1% uranyl acetate for 1 min, dried for 3 h, and visualized on a JEOL JEM 1010 TEM electron microscope equipped with a 2k x 2k AMT CCD camera for digital image acquisition. The sizes for individual EVs were measured using ImageJ software. After calibrating the scale bars, the straight tool was used to measure the diameter.

### Measurement of U87 EVs Size and Concentration Distribution with NTA

EVs purified from 2D and 3D cultures were analyzed using the NanoSight LM 10 instrument (NanoSight Ltd), using an automatic syringe pump for sample injection. The analysis settings were optimized and kept constant between samples, and 1-min videos were recorded to obtain the mean, mode, median, and estimated concentration for each sample (5 videos were analyzed per sample). Sample dilutions for EV isolate samples were adjusted to a final 20 to 100 particles per field of view (1:100–1:1000 dilutions). 1X dPBS was used for cleaning between samples to guarantee there were no carryover particles. The settings were adjusted to camera level 16 and detection threshold 5 for all analyses. Syringe pump speed was set to 50. The data were analyzed using NTA software version 2.3.

### Analysis of EV Membrane Proteins by Multiplex Bead-Based Flow Cytometry Assay

EV samples from 2D and 3D cultures were analyzed with the MACSPlex EV Kit IO (for immuno-oncology) (#130-108-813) (Miltenyi Biotec). EVs were incubated with an antibody-capture bead mixture containing 39 unique fluorescently barcoded capture beads and three detection antibodies (CD9, CD63, CD81). Samples were incubated with rotation and protection from light overnight at room temperature. Further preparation was performed with a 0.2 μm PES filter plate on a vacuum manifold. Flow cytometry acquisition was conducted using the Cytek Aurora (Cytek Biosciences). Kit-included beads were utilized for gate setup; fluorescent multi-peak beads (Cytek QbSure 6-peak beads, B7-10005, lot #: AF01) were acquired for cross-calibration purposes. Flow cytometry files were calibrated to MESF units using FCMPASS software (v4.1.1, https://nano.ccr.cancer.gov/software/) (27), and further analyzed in FlowJo (v10.10.0) and the MPAPASS software (v1.01, https://nano.ccr.cancer.gov/software/) (28). Normalized datasets were constructed for MESF fold-change and comparisons of individual markers. Heatmaps were generated using fold-change normalization to bead plus antibody controls with log_10_ data scaling. For plots comparing MESF of individual EV markers between 2D and 3D EVs, data normalization was performed by background subtraction of unstained control beads for all samples to obtain MESF units above background.

### Analyses of EV Proteins by Silver Staining SDS-PAGE and Western Blotting

A pool containing 10 μl of each EV replicate (2D cell culture and EVs DMSO, TMZ 100, and TMZ 200 μM; 3D cell cultures and EVs DMSO, TMZ 100, and TMZ 200 μM) was prepared and lysed using 10% of the total volume with RIPA Lysis and Extraction Buffer (#89901, ThermoFisher) plus Halt Protease Inhibitor Cocktail (100X) (#78438, ThermoFisher). The mixed solution of pooled EVs plus lysis buffer was vortexed for 1 min. Protein quantification was performed using Micro BCA Protein Assay Kit (#23235, ThermoFisher). For SDS-PAGE, 5 μg of proteins obtained from 2D and 3D cell cultures and 15 μl of 2D and 3D proteins obtained from EVs were mixed with 5 μl of 4X Bolt LDS sample buffer (#B0007, Thermo Fisher) plus DTT (Thermo Fisher). This mixture was heated at 99 °C for 10 min, and electrophoresis was conducted on NuPAGE bis-tris mini protein gels, 4 to 12%, 1.0 to 1.5 mm (#NP0322BOX, Thermo Fisher) at 120V for 90 min. Silver staining was performed using Pierce silver stain kit (#24612, Thermo Fisher) following the manufacturer's instructions. For western blotting, proteins were transferred to an Immun-Blot LF PVDF membrane (#162-0264, Bio-Rad) using a trans-blot turbo transfer buffers and system (Bio-Rad) for 7 min. After transfer, membranes were blocked for 1 h at room temperature with 5% skim milk in 1x PBST buffer, then incubated overnight at 4 °C with the following primary anti-human antibodies: CD44 (HCAM) (1:200, #sc-7946, Santa Cruz Biotechnology), β-catenin (1:200, #sc-7199, Santa Cruz Biotechnology), GAPDH (1:200, #sc-47724, Santa Cruz Biotechnology), β-actin (1:200, #sc-47778, Santa Cruz Biotechnology), and CD81 (1:500, #sc-166029, Santa Cruz Biotechnology). Subsequently, membranes were incubated with the following secondary antibodies: goat anti-rabbit IgG (H + L) highly cross-adsorbed secondary antibody, Alexa Fluor plus 647 (1:1000, #A-21245, Invitrogen), and donkey anti-mouse IgG (H + L) highly cross-adsorbed secondary antibody, Alexa Fluor plus 488 (1:1000, #A-21202, Invitrogen) at room temperature for 1 h. Fluorescence signals were then visualized and documented by a ChemiDoc MP imaging system (Bio-Rad, US).

### Protein Isolation for Proteomic Profiling

After 72 h of TMZ treatment and collecting the media for EV isolation, U87 2D (one T-75 flask, 8 × 10^6^ cells per replicate) and 3D cell cultures (one ULA well, approximately 2885 spheroids per replicate) were harvested by the addition of protein lysis buffer containing 8 M urea, 6 mM TCEP, and 0.05% (w/v) n-dodecyl-β-D-maltoside (DDM) in water. Cell lysates were transferred to 2 ml Eppendorf tubes, vortexed for 1 min, and probe-sonicated for 30 s. For EV protein analysis, 40 μl of each purified EV sample was mixed with 40 μl of the same lysis buffer and vortexed for 1 min. Total protein amount isolated from cells and EV samples was quantified and characterized using μBCA, and SDS-PAGE gels were processed using Coomassie blue and silver staining protocols.

### Experimental Design and Statistical Rationale

Proteomic analyses were performed using a label-free quantitative workflow with biological replication designed to ensure statistical robustness. For each experimental condition, six biological replicates were analyzed. For 3D-spheroid cultures, biological replicates consisted of six independently grown wells from a 6-well plate, while for 2D monolayer cultures, replicates consisted of six independently cultured T225 flasks. Extracellular vesicles and corresponding cellular proteomes were isolated, processed, and analyzed independently for each biological replicate. Biological replicates were chosen rather than technical replication at the LC–MS/MS level to capture biologically meaningful variability between culture conditions and treatments. Statistical comparisons were performed using established differential expression workflows for label-free quantitative proteomics, with appropriate normalization, imputation of missing values, and multiple-testing correction applied to control false discovery rates. The number of biological replicates provided sufficient power to detect consistent and reproducible differences in protein abundance between 2D and 3D culture systems and between treatment conditions, supporting the statistical validity of the reported proteomic findings.

### U87 2D and 3D Proteomic Profiling

#### Sample Preparation

U87 2D and 3D-spheroid cultures and the corresponding EV isolates were digested using the On-Micro Solid-phase Extraction Tip-based (OmSET) method as described previously (29). Briefly, 80 μl of each EV isolate or cell lysate was loaded into a 200 μl pipette tip that was previously packed with three disks of a C18 membrane. Samples lysed as described above were reduced and alkylated with 50 mM TCEP, 10 mM IAA in 30 mM ammonium bicarbonate pH 8 and digested overnight at 45 °C with Lys-C and trypsin. Peptides were eluted into glass LC autosampler inserts (Thermo Fisher Scientific) with 65% ACN in 0.1% FA, dried down using a speedvac, and reconstituted in 5 μl 2% ACN in 0.1% FA.

#### Liquid Chromatography

An in-house bead-packed C18 column was used for analysis. In short, a fused silica capillary (75 μm I.D., 360 μm O.D., Polymicro Technologies, Phoenix, AZ) was pulled to generate an ESI emitter tip of ∼5 μm ID, and the capillary was packed with C18 beads (Dr Maisch, 1.9 μm, 120 Å) to 15 cm in length. An UltiMate 3000 nanoLC system (Thermo Fisher Scientific) was used for nanoflow chromatography. The analytical column was connected to a piece of fused silica transfer tubing (20 μm, 360 μm, 1 m) via a tee union, which was connected via Nanoviper to a column-switching valve. ESI voltage was applied to the tee union to generate nanoESI of the analytes eluting off the column. Samples were loaded at 150 nl/min for 15 min using 1% B. Analytes were eluted from the column at 150 nl/min using a 1 h linear gradient from 1% B to 23% B, where solvent A was 0.1% formic acid (FA) in water and solvent B was 0.1% FA in acetonitrile (ACN). Then, the solvent composition was changed from 23% B to 80% B over 3 min and held for 2 min. Finally, the solvent composition was changed to 1% B over a period of 0.1 min and held for 20 min.

#### Mass Spectrometry

Technical duplicate injections were made using an UltiMate 3000 nanoLC coupled to an Exploris 480 Orbitrap mass spectrometer (Thermo Fisher Scientific). The ion transfer tube temperature was set to 275 °C, and the ESI voltage was set to 1.5 kV using a Nanospray Flex Ion Source. The instrumentation was configured in data-dependent acquisition (DDA) mode using positive polarity. Full MS1 scans were acquired from 375 to 1800 *m/z* at a resolution of 120,000 (at 200 *m/z*) with automatic gain control (AGC) target set to 3 × 10^6^, maximum injection time set to 200 ms, and funnel RF level at 40. The highest intensity peaks in a cycle time of 3 s were selected for higher-energy collisional dissociation (HCD) fragmentation with charge states 2 through 5. The normalized collision energy was set to 30%. MS2 scans were collected at a resolution of 30,000 (at 200 *m/z*), and the isolation window was set to 2 *m/z*. The maximum injection time was set to 500 ms with an AGC target of 1 × 10^6^, and the intensity threshold was kept at 8 × 10^3^. The lowest level for MS2 scanning range was set to 110 *m/z*. Dynamic exclusion was set to 45 s, and isotope exclusion was on.

#### Proteomics Data and Bioinformatics Analysis

Raw files were processed using Proteome Discoverer (version 2.4, Thermo Fisher Scientific) employing the Sequest HT search engine against the UniProtKB/Swiss-Prot human database (Release 2020_01, containing 20,302 sequences). Trypsin was specified as the proteolytic enzyme, allowing up to two missed cleavages. The precursor ion mass tolerance was set to 10 ppm, and the fragment ion mass tolerance to 0.02 Da. Carbamidomethylation of cysteine residues was specified as a fixed modification, while oxidation of methionine and protein N-terminal acetylation were included as variable modifications. Label-free quantification was performed using feature-based quantification with the Match Between Runs functionality enabled, allowing transfer of peptide identifications across LC–MS/MS runs based on accurate mass and aligned retention times. Peptide-spectrum matches were filtered using a target–decoy strategy to control false discovery rates, with an FDR threshold of 1% applied at both peptide and protein levels.

Downstream proteomics data analysis was performed in the R environment (version 4.3.1) using the Differential Expression Proteomics (DEP) package available through Bioconductor, as previously described (30). EV and cellular proteomics datasets were processed and analyzed separately. The raw output tables generated by Proteome Discoverer for cellular and EV proteomes are provided in Supplemental Tables S1 and S2, respectively. As an initial post-processing step, proteins detected in fewer than 20% of samples were excluded from further analysis. The remaining data were normalized using a variance-stabilizing transformation. Missing values were imputed using random draws from a left-shifted Gaussian distribution (shift = 1.8, scale = 0.3), consistent with standard assumptions for missing-not-at-random values in label-free quantitative proteomics.

### *In silico* Validation Using the Cancer Genome Atlas (TCGA)

One hundred 18 highlighted proteins from U-87 cells and extracellular vesicles (EVs), identified through mass spectrometry, were analyzed against a glioblastoma primary tumor RNA database from *The Cancer Genome Atlas* (TCGA). A heatmap and an up/down expression profile were generated using *the University of Alabama at Birmingham Cancer Data Analysis Portal* (UALCAN), available at https://ualcan.path.uab.edu/index.html. Kaplan-Meier survival plots were obtained from *The Human Protein Atlas*, accessible at https://www.proteinatlas.org/.

### Statistical Analysis of the Data

Statistical analyses of TMZ-induced cell death in U87 cells, as well as EV concentration and size measurements by NTA, were performed using two-way ANOVA followed by Šídák’s multiple comparisons test. The data are represented in graphs as means ± SD. The histogram distributions for EVs’ diameters obtained by TEM were analyzed by the Chi-square test. Statistical analyses were performed using GraphPad Prism v. 9, and data were considered significant if *p* < 0.05 (∗), *p* < 0.01 (∗∗), *p* < 0.001 (∗∗∗), and *p* < 0.0001 (∗∗∗∗). For proteomic analysis, protein-wise linear models combined with empirical Bayes statistics were used for the differential enrichment analysis (or differential expression analysis). Differential expression analyses were performed using adjusted *p*-value thresholds tailored to dataset characteristics. A more stringent cutoff (Padj <0.01) was applied to cellular proteomes, which exhibited higher protein coverage and lower missingness, whereas a slightly more permissive threshold (Padj <0.05) was applied to EV proteomes to account for lower complexity and increased stochasticity, while maintaining appropriate control of false discoveries. Besides the visualizations provided by DEP, Volcano and PCA plots were generated in R. Venn diagrams were generated using the web-based tool InteractiVenn (31), available at https://www.interactivenn.net/. For Venn diagram analysis, we considered only proteins present in at least two out of the six replicates in each group. Heatmaps of GBM markers and methyltransferases were generated using the Morpheus tool (https://software.broadinstitute.org/morpheus/), with Euclidean distance applied for both rows and columns. Functional enrichment analysis was performed using Gene Ontology (GO) biological process annotations with Benjamini–Hochberg correction (Padj <0.05). Protein–protein interaction networks were analyzed using the STRING database with a high-confidence interaction score (≥0.7).

## Results

### Temozolomide (TMZ) Increases Cell Death in U87 2D and 3D-Spheroid cell Culture Models

**Fig. 1:**
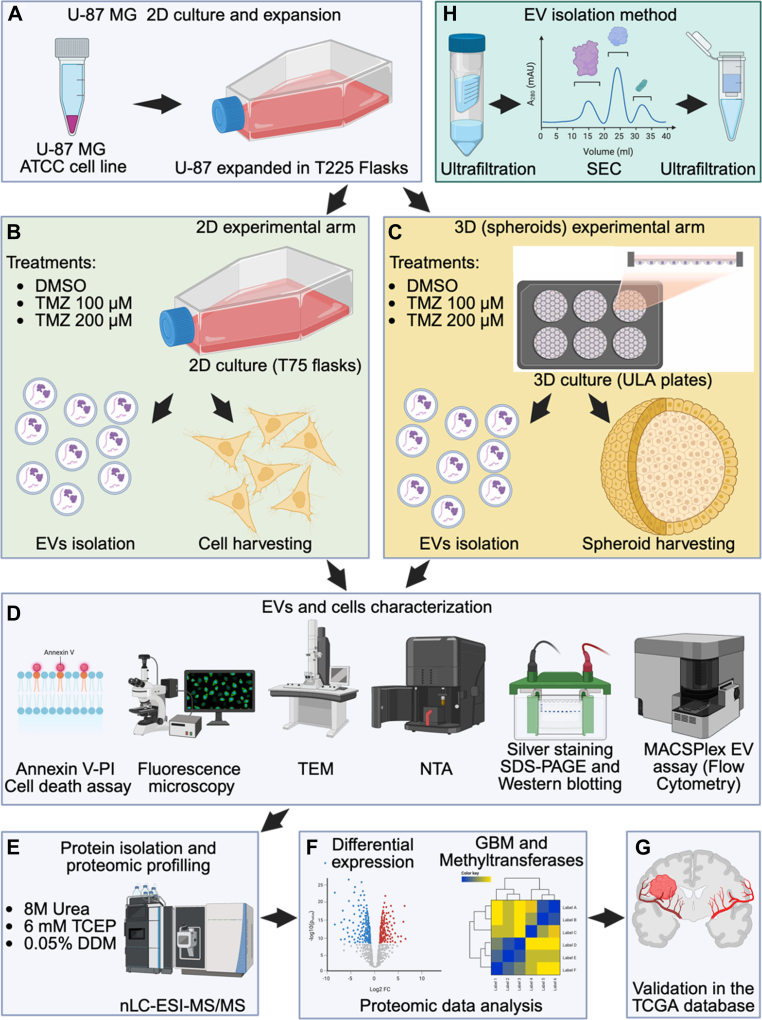
**Experimental workflow overview.** This schematic illustrates the experimental design used to profile extracellular vesicles (EVs) derived from U87 glioblastoma cells cultured under 2D monolayer and eroid conditions. *A*, expansion of U-87 MG glioblastoma cells in T225 flasks prior to experimental setup. *B*, 2D experimental arm: cells cultured in T75 flasks and treated with DMSO, temozolomide (TMZ) 100 μM, or TMZ 200 μM, followed by EV isolation and cell harvesting. *C*, 3D-spheroid experimental arm: cells seeded in ultra-low attachment (ULA) plates and treated as in (*B*), with subsequent EV isolation and spheroid harvesting. *D*, EV and cell characterization by Annexin V/PI cell death assay, fluorescence microscopy, transmission electron microscopy (TEM), nanoparticle tracking analysis (NTA), silver-stained SDS-PAGE, western blotting, and MACSPlex EV flow cytometry assay. *E*, protein extraction from EVs and cells, followed by proteomic profiling using nano-liquid chromatography tandem mass spectrometry (nLC-ESI-MS/MS). *F*, differential expressions and functional analysis, including heatmap clustering and identification of GBM-associated protein families (e.g., methyltransferases). *G*, cross-referencing of selected proteins with The Cancer Genome Atlas (TCGA) for prognostic relevance. *H*, EV isolation pipeline combining ultrafiltration and size-exclusion chromatography (SEC) for purification of vesicles from conditioned media.

To understand the effect of TMZ on the proteome of U87 cell lines cultured under different conditions (2D and 3D) and on their respective EV proteomes, we treated the cell cultures with clinically relevant concentrations of TMZ (100 and 200 μM), or DMSO control. The experimental workflow is shown in Figure 1. Spheroid formation (i.e., 3D cell cultures), enabled using ultra-low attachment (ULA) plates, was monitored daily for 5 days by phase-contrast microscopy. The results revealed spheroids of approximately 200 μm in diameter, on average (Supplemental Fig. S1). To evaluate the effect of TMZ on cell apoptosis and necrosis, U87 2D cell cultures (Supplemental Fig. S2*A*) and 3D-spheroids (Supplemental Fig. S2*B*) were treated with 100 and 200 μM of TMZ for 72 h, followed by an Annexin-V/PI assay. The results demonstrated a dose-dependent significant increase in apoptosis and necrosis in 2D cell culture (*p* < 0.05 and *p* < 0.0001, respectively; Supplemental Fig. S2*C*). In the case of 3D-spheroids, TMZ treatments promoted less apoptosis but increased significantly necrosis in a dose-dependent manner (*p* < 0.0001; Supplemental Fig. S2*D*). For better visualization of the U87 cells and spheroids, images were acquired using a 20X0.75 PlanFluorite objective (Supplemental Fig. S3, top and bottom panels). We also tested the effect of increased TMZ range (from 50 to 1600 μM) for 72 h on larger U87 3D spheroids (approximately 1 mm in diameter) generated using a 96-well ULA plate. The results of the live/death cell staining showed a dose-dependent increase in cell death (Supplemental Fig. S4). The images also indicate that the center of the spheroids contained fewer dead cells (darker color), possibly due to decreased diffusion of the TMZ through the cells.

### U87 3D-Spheroids Release More EVs with Smaller Sizes compared to 2D Cultures

**Fig. 2:**
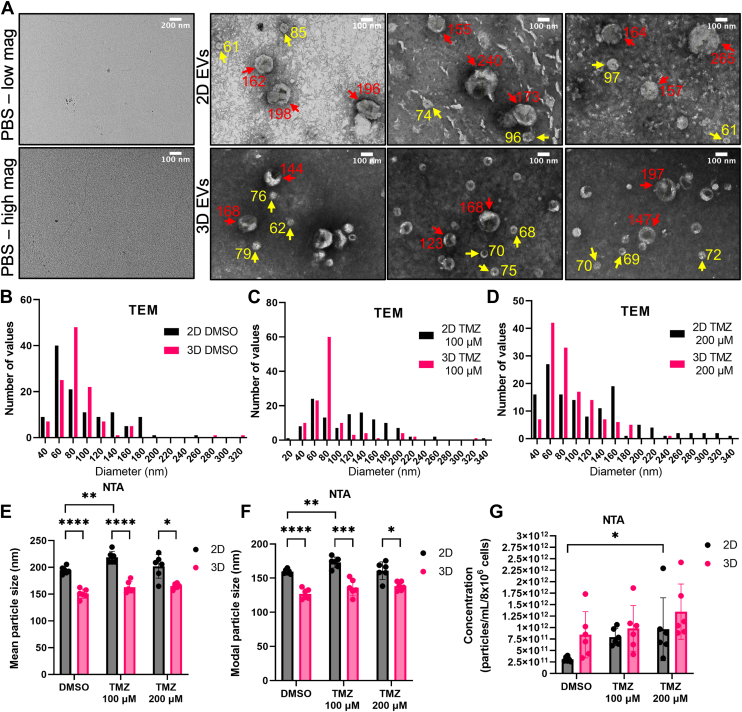
**U87 cells release extracellular vesicles (EVs) with distinct size and abundance profiles in 2D vs. 3D cultures.***A*, transmission electron microscopy (TEM) revealed cup-shaped EVs ranging from 40 to 300 nm in diameter. Both small (*yellow arrows*) and large (*red arrows*) EVs were observed, with a notable increase in large EVs in TMZ-treated 2D cultures compared to 3D-spheroids. *B–D*, size distribution histograms from nanoparticle tracking analysis (NTA) show distinct profiles between 2D and 3D EVs across treatments (DMSO, TMZ 100 μM, TMZ 200 μM). Statistically significant differences in size distribution were observed between 2D and 3D EVs for each condition (Chi-square test, *p* < 0.05). *E*, mean EV diameters were significantly smaller in 3D-derived EVs compared to 2D across all treatments (Two-way ANOVA, *p* < 0.05 to *p* < 0.0001). *F*, modal diameters followed a similar trend, with smaller modal sizes in EVs from 3D cultures. *G*, when normalized to cell number, 3D-spheroids released more EVs than 2D cultures, although this difference was not statistically significant (*p* > 0.05). In contrast, 2D cultures treated with 200 μM TMZ released significantly more EVs compared to DMSO controls (*p* < 0.05).

After validating the effects of TMZ on cell death, we isolated EVs from 2D and 3D cultures under each treatment condition. Initially, we labeled purified EVs with a lipophilic dye, CellMask Red, and observed them under a fluorescence microscope. CellMask-positive particles were visible in DMSO- and TMZ-treated EV samples, from both 2D and 3D cultures, but absent in the blank or void controls (Supplemental Fig. S5). To further characterize these particles, negative staining TEM was performed, revealing small and large cup-shaped structures in the size range of 60 to 300 nm for both 2D and 3D cultures. The diameter of small and large EVs were measured and labeled for several representative EVs in the presented images as yellow and red, respectively (Fig. 2*A*). Next, we measured the diameter of individual particles and found that 2D cultures exhibited a bimodal distribution of EVs, comprising small EVs (60 nm) and large EVs (140–160 nm) under both DMSO- and TMZ-treated conditions. In contrast, 3D cultures showed a normal-like unimodal distribution with a peak in the small EV range (60–80 nm) under both DMSO- and TMZ-treated conditions. The differences in the size distribution of EVs obtained by TEM from 2D and 3D cultures (Fig. 2, *B*–*D*) were statistically analyzed using the Chi-square test and found to be significant (*p* < 0.05).

As an orthogonal method to compare EV size distributions, we utilized NTA. The results indicated that 2D cultures released EVs with larger mean (Fig. 2*E*) and modal sizes (Fig. 2*F*) compared to 3D cultures (*p* < 0.0001), likely due to the higher number of larger EVs (140–160 nm) released by 2D cultures. Additionally, using NTA to count the number of particles released per 8 × 10^6^ cells, we observed that while 3D-spheroid cultures consistently seem to release more EVs than 2D cultures, the differences did not reach statistical significance (*p* > 0.05). Moreover, TMZ treatment at 200 μM significantly increased the EV release (*p* < 0.05) compared to DMSO in 2D cultures (Fig. 2*G*).

### Analysis of 2D and 3D EV Protein Markers by Flow Cytometry and Western Blotting

**Fig. 3:**
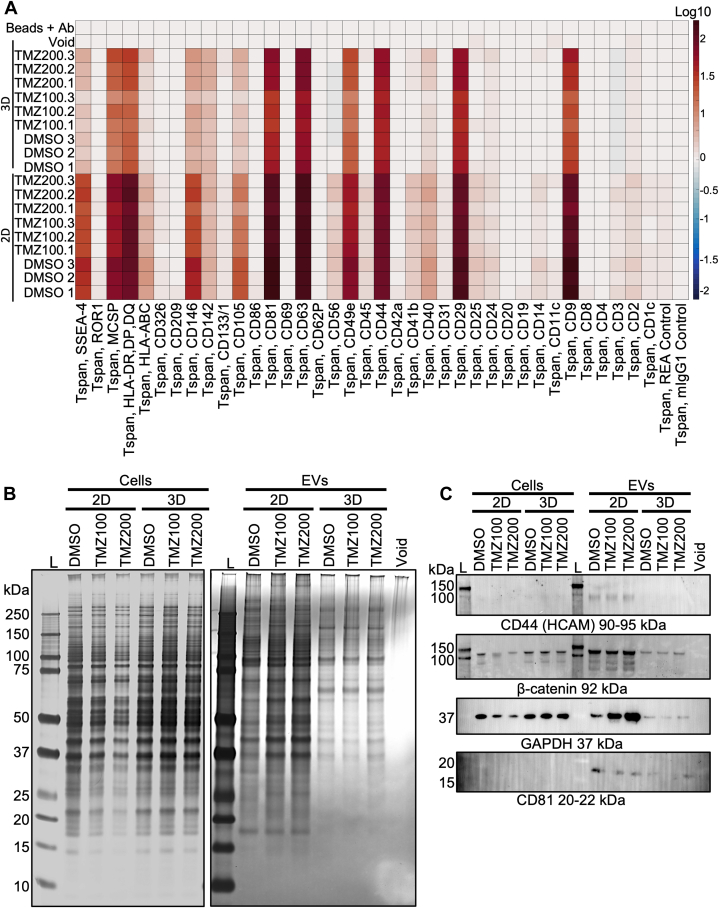
**Characterization of EV protein profiles from U87 2D and 3D cultures.***A*, multiplex flow cytometry using the MACSPlex EV kit was used to profile surface markers of EVs derived from U87 cells cultured under 2D and 3D conditions. CD44 was consistently detected and enriched across conditions, particularly in TMZ-treated samples. The integrins CD29 and CD49e—subunits of the α5β1 integrin heterodimer—were robustly expressed in both 2D and 3D EVs, highlighting their potential role in EV-mediated adhesion and signaling. *B*, silver-stained SDS-PAGE showing global protein profiles of cell lysates and EVs from 2D and 3D cultures. Distinct banding patterns reflect culture-specific proteomic signatures and successful protein isolation. *C*, Western blot analysis confirms the presence of established EV-associated proteins, including CD81 and CD44. GAPDH, were used as controls and were detected predominantly in cell lysates, supporting the purity of the EV preparations and absence of major cellular contamination.

Following the characterization of intact EVs by fluorescence microscopy, TEM, and NTA, we performed a multiplex bead-based flow cytometry assay and western blotting to measure potential shifts in the antigen composition promoted by TMZ treatments. The MACSPlex Flow Cytometry assay affords detection of 37 EV surface epitopes. We utilized three replicates of purified EV samples, each derived from separate T75 flasks (2D culture) or ULA wells (3D-spheroid culture) under different treatment conditions (DMSO or TMZ). Besides high reproducibility of the signal intensities between replicate EV isolations, the flow cytometry analysis revealed an enrichment of tetraspanins CD9, CD63, and CD81, as well as the three MHC class II isotypes HLA-DR, DP, and DQ. Additionally, other highly abundant EV membrane proteins, including CD44 (HCAM), CD29, CD49e, CD146, CD105, melanoma-associated chondroitin sulfate proteoglycan (MCSP), and stage-specific embryonic antigen-4 (SSEA-4), were identified. Proteins detected in EVs at lower abundances included HLA-ABC, CD142, and CD40. Notably, CD56 (Neural Cell Adhesion Molecule, NCAM) was exclusively identified in 2D culture-derived samples (Fig. 3*A*). We further analyzed the total protein content of parent U87 cells and U87-derived EVs cultured under both 2D and 3D conditions using silver-stained SDS-PAGE. For this analysis, we prepared a single pooled sample comprising six replicates for each condition. The silver-stained SDS-PAGE revealed well-defined protein bands ranging from approximately 15 kDa to above 250 kDa. The protein repertoire of parent U87 cell lysates (both 2D and 3D) appeared richer with diverse proteins compared to that of the EVs. Interestingly, in U87 cells, the intensity of protein bands in the 2D TMZ 200 μM treatment was fainter compared to DMSO and TMZ 100 μM treatments. Conversely, in 2D EVs, the band intensity increased with higher TMZ concentrations (Fig. 3*B*). Western blot analysis revealed CD44 (HCAM) exclusively in 2D EVs. β-catenin was detected across all samples, with altered band patterns in 2D cell cultures in response to increasing TMZ doses, suggesting differential abundance of post-translational modifications. The increased abundance of GAPDH in 2D EVs is particularly interesting, as it reflects the opposite trend observed in 2D models, an increase in abundance with TMZ treatment in 2D EVs versus a decrease in the 2D cultured cells. CD81 appeared as faint bands and was detected exclusively in EV preparations (Fig. 3*C*).

### TMZ Treatment Induces Distinct Proteomic Adaptations in U87 2D and 3D-Spheroid Cell Cultures

**Fig. 4:**
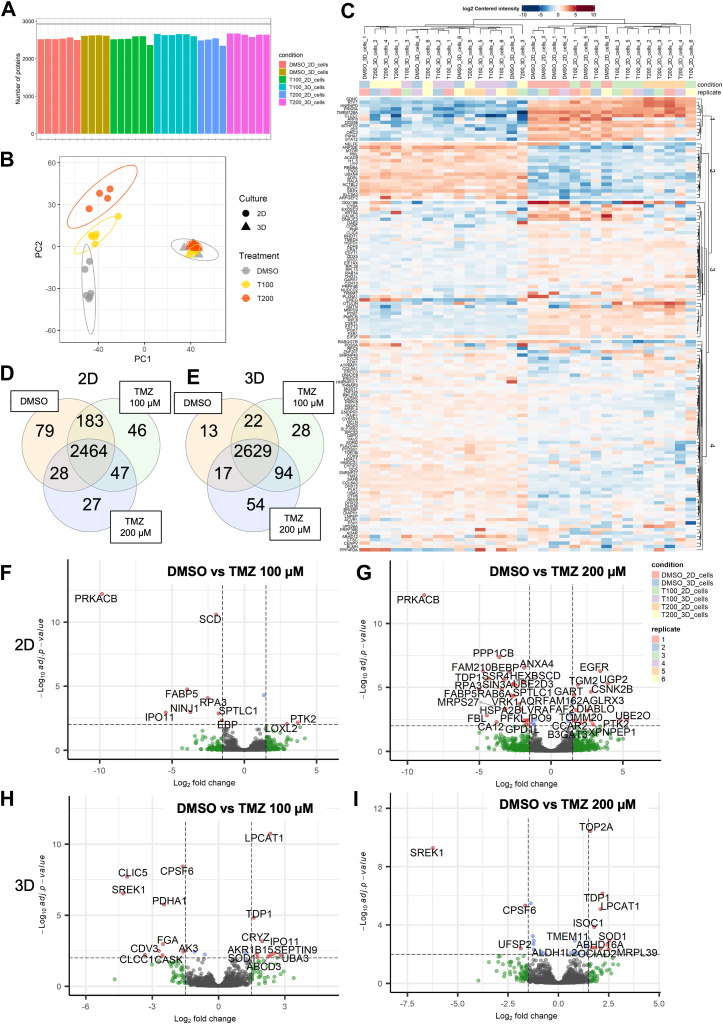
**Temozolomide (TMZ) induces distinct proteomic remodeling in U87 Cells Cultured in 2D and 3D.***A*, Bar plot showing the total number of proteins identified per sample across all conditions, with a mean of ∼2500 proteins, demonstrating high coverage and technical consistency. *B*, principal component analysis (PCA) of global proteomic profiles showing distinct separation along PC1 between 2D and 3D cultures, and along PC2 between DMSO and TMZ treatments in 2D, indicating that both culture dimensionality and drug exposure influence proteomic variance. *C*, Heatmap of the top 133 most abundant proteins across all conditions, illustrating overall expression trends and sample clustering. These proteins highlight the major proteomic differences between 2D and 3D cultures and across treatments. *D, E*, Venn diagrams of proteins identified in U87 cells under each treatment condition for 2D (*D*) and 3D (*E*), illustrating shared and unique protein identities in response to TMZ. *F–I*, Volcano plots showing significantly differentially expressed proteins (adjusted *p* < 0.01, |log_2_FC| > 1.5) between DMSO and TMZ-treated cells at 100 μM and 200 μM in 2D (*F*, *G*) and 3D cultures (*H*, *I*). Notable proteins involved in stress response, metabolism, and translation are highlighted. Overall, TMZ induced more pronounced proteomic shifts in 2D cultures, whereas 3D spheroids exhibited a more restricted remodeling pattern, consistent with enhanced resistance.

To investigate TMZ-induced proteomic changes in U87 cells and their derived extracellular vesicles (EVs), we initially performed bottom-up mass spectrometry-based proteomic analysis on cells cultured under 2D and 3D-spheroid conditions. Data processing, including normalization, missing value imputation, and global protein abundance heatmaps, is presented in Supplemental Fig. S6. On average, approximately 2500 proteins were identified and quantified per sample, with slightly higher proteome coverage observed in 3D-spheroid cultures (Fig. 4*A*). Principal component analysis (PCA) revealed clear separation between 2D and 3D proteomic profiles along PC1, indicating substantial differences driven by culture architecture (Fig. 4*B*). In response to TMZ, 2D cell cultures exhibited a dose-dependent shift in proteomic profiles, whereas 3D-spheroid cultures showed a more limited response, with separation observed primarily at the highest TMZ concentration. These findings suggest that 3D cultures display a more stable or buffered proteomic state under TMZ exposure. This pattern was further supported by independent PCA analyses of each culture system (Supplemental Fig. S7). Unsupervised clustering of the most abundant proteins confirmed a strong segregation between 2D- and 3D-derived proteomes (Fig. 4*C*), highlighting fundamental differences in cellular organization and functional states between the two models.

Analysis of proteins uniquely induced by TMZ revealed 47 proteins specific to 2D cultures and 94 proteins unique to 3D-spheroids, consistently detected across both TMZ concentrations but absent in DMSO-treated controls (Fig. 4, *D* and *E*). Gene Ontology (GO) analysis indicated that proteins uniquely detected in 2D cultures were associated with activation of DNA damage checkpoint pathways (e.g., XPC, ERCC3, CDK2), consistent with a canonical response to TMZ-induced genotoxic stress. In contrast, proteins uniquely detected in 3D-spheroid cultures were enriched in nucleotide biosynthetic processes (e.g., IMPDH2, PRPS2), suggesting enhanced metabolic support for DNA repair and replication. Together, these results indicate that while 2D cell cultures predominantly activate acute DNA damage responses, 3D-spheroid cultures exhibit a more adaptive phenotype characterized by metabolic reprogramming to sustain nucleotide availability, a feature closely associated with glioblastoma survival and resistance to alkylating chemotherapy. Differential expression analysis further highlighted distinct TMZ responses between the two systems. In 2D cultures, a relatively limited number of proteins were altered at 100 μM TMZ, whereas a broader proteomic shift was observed at 200 μM (Fig. 4, *F* and *G*). These changes were primarily associated with lipid metabolic pathways, particularly monounsaturated fatty acid biosynthesis (e.g., SCD, FABP5, SPTLC1). In the context of glioblastoma, this remodeling is highly relevant, as lipid metabolism supports membrane synthesis, signaling platform organization, and extracellular vesicle biogenesis, all of which contribute to cellular adaptation and survival under chemotherapeutic stress. In contrast, 3D-spheroid cultures displayed a more restrained differential response, with fewer proteins altered even at higher TMZ concentrations (Fig. 4, *H* and *I*). GO term enrichment analysis of these proteins revealed enrichment in processes related to the regulation of phosphatidylcholine biosynthesis (e.g., LPCAT1), indicating modulation of membrane lipid composition and dynamics. In glioblastoma, such lipid remodeling is linked to membrane fluidity, oxidative stress management, and DNA repair capacity, suggesting that 3D-spheroids adopt a protective metabolic state that supports survival and increased resistance to TMZ treatment. The complete list of differentially expressed proteins in 2D and 3D cell cultures is provided in Supplemental Table S3.

### TMZ Treatment Induces Distinct Proteomic Adaptations in EVs Secreted by U87 2D and 3D-Spheroid Cultures

**Fig. 5:**
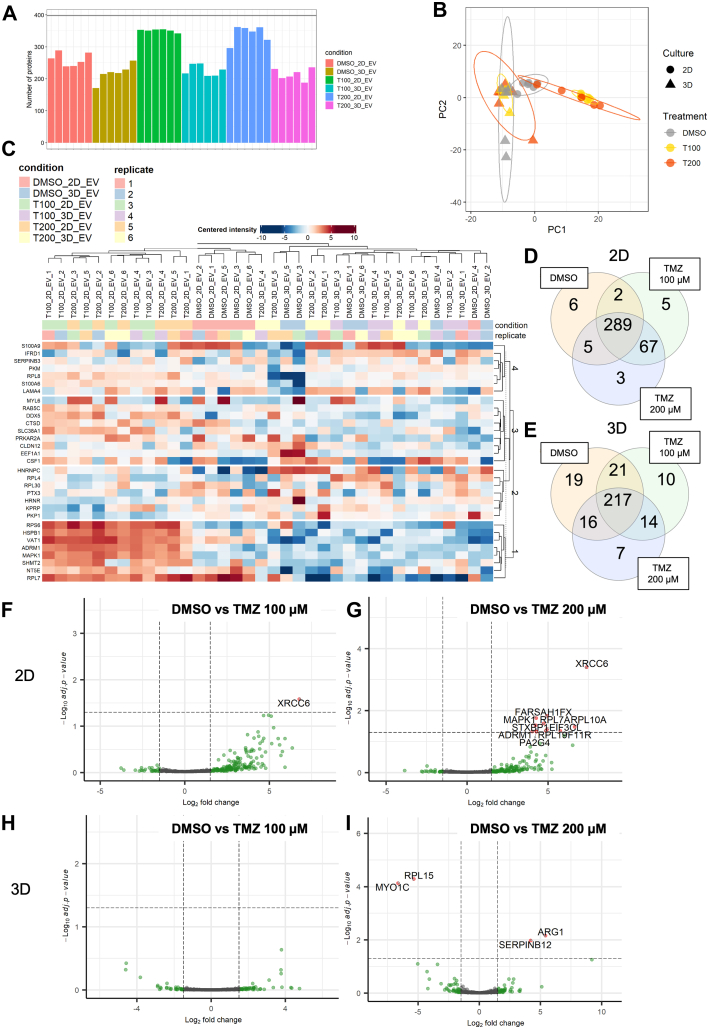
**TMZ Alters the Proteome of EVs from U87 Cells Cultured in 2D and 3D Conditions.***A*, approximately 250 proteins were identified per sample, representing the core proteome of extracellular vesicles (EVs) released from U87 cells cultured under 2D and 3D conditions. *B*, principal component analysis (PCA) revealed partial separation between 2D and 3D EVs along PC1, with clearer separation observed between DMSO- and TMZ-treated groups, particularly within the 2D EVs. *C*, heatmap of the 133 most abundant EV proteins (not necessarily differentially expressed) revealed clustering based on both culture type and treatment, demonstrating the selective enrichment of protein subsets under genotoxic stress. *D–E*, Venn diagrams illustrate the overlap and unique protein sets in EVs derived from 2D and 3D cultures across DMSO, TMZ 100 μM, and TMZ 200 μM conditions. *F–I*, Volcano plots of differentially expressed proteins (Padj < 0.05, |LFC| > 1.5) between DMSO and TMZ-treated EVs show distinct proteomic shifts in response to treatment in 2D (*F*–*G*) and 3D (*H*–*I*) cultures. While 2D-derived EVs exhibit stronger treatment-specific signatures, EVs from 3D-spheroids show more limited proteomic remodeling.

Following the proteomic profiling of U87 parental cells, we isolated and analyzed EVs derived from 2D and 3D-spheroid cultures using LC-MS-based bottom-up proteomics. Data processing, including normalization, missing value imputation, and global protein abundance heatmaps, is presented in Supplemental Fig. S8. Despite using lower input volumes for EV isolation, we identified between 200 and 350 proteins per sample, with slightly higher protein coverage in EVs derived from 2D cultures (Fig. 5*A*). Principal component analysis (PCA) revealed clear separation between EV proteomes derived from 2D and 3D cultures along PC1, mirroring the differences observed in parental cells (Fig. 5*B*). In response to TMZ, EVs from 2D cultures exhibited a dose-dependent shift in proteomic profiles, whereas EVs derived from 3D-spheroids showed minimal separation across treatment conditions. This pattern was further supported by independent PCA analyses of each culture system (Supplemental Fig. S9), indicating that EV cargo from 3D cultures is less dynamically altered in response to TMZ.

Unsupervised clustering of the most abundant EV proteins further confirmed a clear distinction between 2D- and 3D-derived EV proteomes (Fig. 5*C*), highlighting differences in EV composition driven by the cell culture architecture. Analysis of proteins uniquely released in EVs following TMZ treatment identified 67 proteins specific to EVs from 2D cultures and 14 unique to EVs from 3D-spheroids, consistently detected across both TMZ concentrations but absent in DMSO-treated controls (Fig. 5, *D* and *E*). GO term enrichment analysis revealed that proteins uniquely present in 2D-derived EVs were associated with cytoplasmic translation (e.g., RPL7A, RPL19, EIF3CL), suggesting selective export of translational machinery components. In contrast, proteins unique to 3D-derived EVs were linked to regulation of nuclear architecture and organization, indicating distinct mechanisms of intracellular adaptation reflected in EV cargo. Notably, ATP-binding cassette sub-family E member 1 (ABCE1), a regulator of ribosome recycling and translation, was detected in EVs from both culture systems under TMZ treatment, suggesting a shared component of EV-mediated stress response. Differential protein abundance analysis further highlighted marked differences between the two systems. In EVs derived from 2D cultures, TMZ treatment induced the enrichment of proteins associated with DNA damage response, translational regulation, and signaling pathways (e.g., XRCC6, MAPK1, and EIF3CL), with a stronger effect observed at 200 μM (Fig. 5, *F* and *G*). GO analysis indicated enrichment in processes related to the regulation of cell–cell interaction and membrane dynamics. Although annotated as negative regulation of myoblast fusion, the contributing proteins are functionally linked to cytoskeletal organization, vesicle trafficking, and intercellular communication (e.g., STXBP1, F11R, and ADRM1). In the context of glioblastoma, these processes are closely associated with EV-mediated signaling, tumor cell invasion, and remodeling of the tumor microenvironment, suggesting that TMZ promotes the release of EVs capable of modulating intercellular interactions and stress adaptation. In contrast, EVs derived from 3D-spheroid cultures exhibited a more limited proteomic response to TMZ. No significant changes were observed at 100 μM, and only a small subset of proteins was altered at 200 μM (Fig. 5, *H* and *I*). These proteins were associated with processes related to immune regulation and stress adaptation, including modulation of arginine metabolism and protease inhibition (e.g., ARG1 and SERPINB12), alongside changes in cytoskeletal and translational components (e.g., MYO1C and RPL15). GO analysis indicated enrichment in immune-related pathways, suggesting that EVs derived from 3D cultures may contribute to modulation of the immune microenvironment. In glioblastoma, such EV-mediated signaling is consistent with mechanisms of immune evasion and metabolic adaptation, supporting tumor survival under chemotherapeutic stress. The complete list of differentially abundant EV proteins isolated from 2D and 3D cultures under the applied treatments is provided in Supplemental Table S4.

### Proteome Profiling of U87-Derived EVs and Their Parental Cells Reveals EV-specific Proteins Associated with Sorting, Packaging, and Release

After characterizing the proteomes of U87 cells and EVs in response to TMZ, we analyzed the specific proteins identified in EVs compared with their parent cells. On average, 82.6% (min. 79.4%; max 85.8%) of the EVs' content overlapped with their matched cell sample, corresponding to around 8.6% (min 5.3%; max 12.1%) of the protein found in the cells. Comparing DMSO-treated 2D cell cultures and their released EVs, we identified 2496 unique proteins in the parental cells, 258 shared proteins between EVs and parental cells, and 44 unique proteins identified only in EVs (Supplemental Fig. S10*A*). For DMSO-treated 3D-spheroid cells and their released EVs, a similar number of shared and unique proteins were identified. We detected 2446 unique proteins in the parental cells, 235 shared proteins between EVs and parental cells, and 38 unique proteins identified only in 3D culture-derived EVs (Supplemental Fig. S10*B*). Functional annotation of these EV-specific proteins revealed enrichment in processes related to membrane organization, immune signaling, and metal ion homeostasis (e.g., MFGE8, HLA-A, SLC30A1, and NCAM1). GO term enrichment analysis further highlighted the enhancement in negative regulation of zinc ion transmembrane transport, suggesting that EVs may contribute to the regulation of metal ion balance and microenvironmental homeostasis under basal conditions. In glioblastoma, such processes are closely linked to oxidative stress regulation, signaling, and tumor–microenvironment interactions. To assess the impact of TMZ on EV cargo selection, we combined proteins identified in both 100 μM and 200 μM treatment conditions and compared EVs with their parental cells. In TMZ-treated 2D cultures, we identified 2500 proteins unique to parental cells, 322 shared proteins, and 52 proteins exclusively detected in EVs (Supplemental Fig. S10*C*). In TMZ-treated 3D-spheroid cultures, 2612 proteins were unique to parental cells, 257 were shared, and 41 proteins were uniquely detected in EVs (Supplemental Fig. S10*D*). Notably, the core set of highly abundant EV-associated proteins observed under control conditions was preserved, following TMZ treatment, indicating maintenance of fundamental EV biogenesis pathways. In EVs derived from TMZ-treated 2D cultures, a subset of proteins was uniquely enriched compared to both parental cells and EVs from DMSO-treated cultures. These proteins were primarily associated with vesicle trafficking, translation regulation, and proteostasis (e.g., VAMP5, EIF3CL, DYNC1LI2, SVIP, GPRC5A, and TRMT5). GO term analysis revealed enrichment in processes related to the negative regulation of the VCP–NPL4–UFD1 AAA ATPase complex assembly, a key pathway involved in protein quality control and ubiquitin-dependent degradation. In the context of glioblastoma, this suggests that TMZ promotes the selective export of proteins involved in proteostasis and intracellular trafficking via EVs, potentially alleviating intracellular stress and contributing to adaptive survival mechanisms. In contrast, EVs derived from TMZ-treated 3D-spheroid cultures exhibited a more limited set of uniquely enriched proteins, with a notable association with stress response and protease inhibition pathways (e.g., SERPINB12). This pattern is consistent with a more constrained but functionally targeted EV response, reflecting the adaptive phenotype of 3D-spheroids. In glioblastoma, such EV-mediated processes may contribute to the establishment of a protective microenvironment through modulation of extracellular proteolysis, immune signaling, and cellular stress responses.

### The Effect of 2D and 3D Cell Culture Methods on the Proteomes of U87 Cells and Secreted EVs

To assess how culture architecture influences proteomic profiles, we compared U87 cells and their secreted EVs under 2D and 3D-spheroid conditions using DMSO-treated samples. At the cellular level, we identified 2538 proteins shared between 2D and 3D cultures, along with 216 proteins unique to 2D cell cultures and 143 proteins unique to 3D-spheroids (Supplemental Fig. S10*E*), indicating substantial overlap alongside distinct condition-specific proteomic signatures. Proteins uniquely detected in 2D cultures were primarily associated with nucleotide metabolism, proliferation, and RNA processing (e.g., IMPDH2, PRPS2, MKI67, and EIF3G). GO analysis revealed enrichment in negative regulation of nitric oxide–cGMP-mediated signal transduction, suggesting that monolayer cultures preferentially engage signaling regulatory pathways linked to proliferation and intracellular communication. In glioblastoma, these pathways are closely associated with rapid cell growth and signaling plasticity. In contrast, proteins uniquely detected in 3D-spheroid cultures were associated with metabolic regulation, intracellular trafficking, and stress adaptation (e.g., RPS6KA1, SQSTM1, VASP, and YIF1A). GO analysis identified enrichment in brown fat cell proliferation; however, functional interpretation of the contributing proteins indicates activation of pathways related to metabolic reprogramming, autophagy, and cytoskeletal remodeling. These processes are consistent with a phenotype characterized by enhanced metabolic plasticity, survival signaling, and structural adaptation, which are hallmarks of glioblastoma progression and therapeutic resistance. We next compared EV proteomes derived from 2D and 3D cultures. A total of 255 proteins were shared between EVs from both conditions, with 47 proteins uniquely detected in 2D-derived EVs and 18 unique to 3D-spheroid-derived EVs (Supplemental Fig. S10*F*), indicating that EV cargo composition is also strongly influenced by culture architecture. EVs derived from 2D cultures were enriched in proteins associated with signal transduction, extracellular matrix interaction, and stress response (e.g., GNAI2, RAP1B, COL1A2, and HSPA1B). GO analysis indicated enrichment in protein heterotrimerization, reflecting activation of G protein signaling pathways and protein complex assembly. In glioblastoma, these processes are closely linked to tumor cell communication, invasion, and interaction with the tumor microenvironment, suggesting that EVs from 2D cultures may promote signaling-driven cellular responses. In contrast, EVs derived from 3D-spheroid cultures were enriched in proteins associated with vesicle trafficking, proteostasis, and metabolic adaptation (e.g., COPB2, VCP, PSMB1, and NRAS). GO analysis revealed enrichment in flavin adenine dinucleotide (FAD) catabolic processes; however, the contributing proteins are functionally linked to protein quality control, intracellular transport, and translational regulation. These findings suggest that EVs from 3D cultures support metabolic rewiring and protein turnover, processes that are critical for glioblastoma adaptation and resistance to stress. Functional enrichment analysis using STRING further supported these distinctions. Proteins enriched in EVs from 2D cultures clustered in pathways related to extracellular vesicle biogenesis and vesicle-mediated transport, consistent with active secretion and intercellular communication (Supplemental Fig. S10*G*). In contrast, proteins enriched in EVs from 3D-spheroids were associated with desmosome-related pathways, reflecting enhanced cell–cell adhesion and structural organization. Given that desmosomes contribute to mechanical cohesion through cadherin-based junctions linked to intermediate filaments, their enrichment in 3D EVs is consistent with the tighter cellular architecture of spheroids and may reflect ongoing adhesion remodeling under these conditions (Supplemental Fig. S10*H*).

### Analysis of GBM Genetic Markers and Methyltransferases

**Fig. 6:**
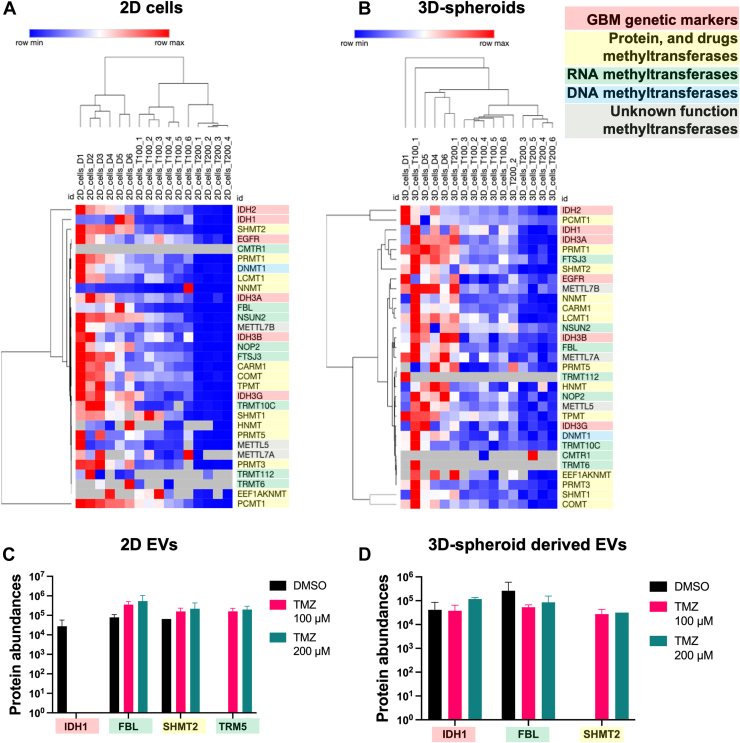
**Expression Patterns of Methyltransferases and GBM Markers in U87 Cells Cultured in 2D and 3D Conditions.** Heatmaps illustrate the relative protein abundance of selected methyltransferases and established glioblastoma (GBM) markers in (*A*) 2D monolayer cultures and (*B*) 3D-spheroid cultures of U87 cells under different treatments (DMSO, TMZ 100 μM, and TMZ 200 μM). Proteins were grouped by functional category, including RNA and DNA methyltransferases, protein/drug-targetable methyltransferases, and GBM-associated genetic markers. Differences in expression between 2D and 3D cultures highlight the influence of tumor architecture on methylation-related pathways and GBM phenotype. *C–D*, Bar graphs show the normalized intensity of selected proteins (IDH1, FBL, SHMT2, and TRM5), revealing distinct modulation in response to temozolomide treatment and 3D organization, suggesting their potential relevance in drug resistance and glioma adaptation.

Since the primary cytotoxic mechanism of temozolomide (TMZ) involves the methylation of DNA bases—particularly at the O^6^ position of guanine—leading to DNA mismatches, strand breaks, and ultimately cell death, we examined the protein levels of 25 endogenous methyltransferases potentially involved in epigenetic regulation and therapy resistance in response to TMZ. In addition, we analyzed the protein levels of common GBM genetic markers, including epidermal growth factor receptor (EGFR) and several enzymes from the isocitrate dehydrogenase (IDH) family: IDH1 (NADP, cytoplasmic), IDH2 (NADP, mitochondrial), IDH3A, IDH3B, and IDH3G (all NAD, mitochondrial subunits). We color-coded the identified GBM genetic markers (red), protein and drug methyltransferases (yellow), RNA methyltransferases (green), DNA methyltransferases (blue), and methyltransferases of unknown function (gray). In both 2D and 3D-spheroid cell cultures, we generally observed a dose-dependent decrease in the levels of these proteins following TMZ treatment (Fig. 6, *A* and *B*, respectively). In contrast, EVs derived from 2D cell cultures showed increased levels of several proteins of interest, including rRNA 2′-O-methyltransferase fibrillarin (FBL), serine hydroxy methyltransferase, mitochondrial (SHMT2), and tRNA (guanine(37)-N(1))-methyltransferase (TRM5) (Fig. 6*C*). For EVs derived from 3D-spheroids, we observed increased levels of IDH1 and SHMT2, while FBL remained stable across TMZ treatments (Fig. 6*D*).

### Mapping the Highlighted Proteins to the Cancer Genome Atlas (TCGA) Data

**Fig. 7:**
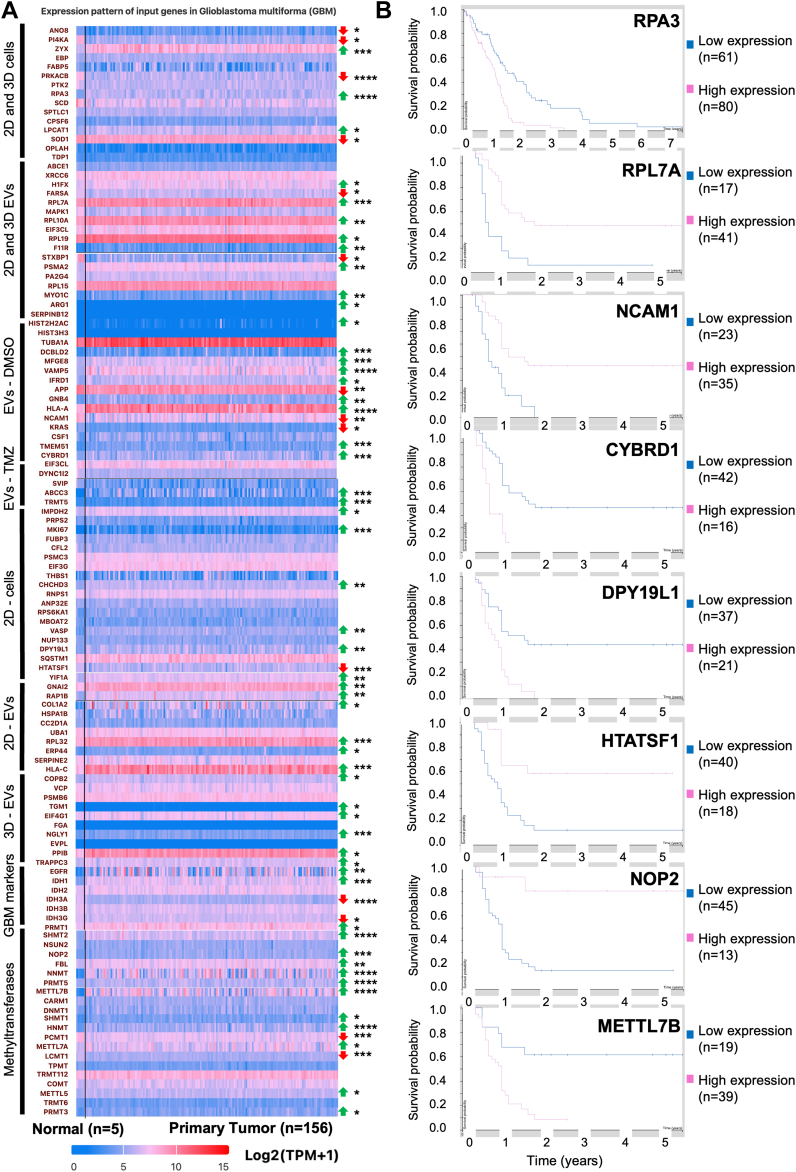
**Gene expression and prognostic relevance of EV- and cell-derived proteins in glioblastoma.***A*, heatmap showing transcript levels (Log_2_TPM+1) of genes corresponding to proteins identified in this study, analyzed using RNA-seq data from The Cancer Genome Atlas (TCGA) for glioblastoma (GBM) tumor samples (n = 156) and normal brain tissue (n = 5). Green and red arrows indicate significantly upregulated and downregulated genes in GBM compared to normal brain, respectively, with statistical significance denoted as *p* < 0.05 (∗), *p* < 0.01 (∗∗), *p* < 0.001 (∗∗∗), and *p* < 0.0001 (∗∗∗∗). *B*, among the 68 differentially expressed genes, high expression of four genes—RPA3 (*p* = 0.000038), CYBRD1 (*p* = 0.00020), DPY19L1 (*p* = 0.00036), and METTL7B (*p* = 0.00066)—was associated with unfavorable prognosis. In contrast, elevated expression of RPL7A (*p* = 0.000093), NCAM1 (*p* = 0.00015), HTATSF1 (*p* = 0.00040), and NOP2 (*p* = 0.00052) correlated with improved overall survival in GBM patients.

Next, we compared the 118 above highlighted proteins—identified across all our datasets (2D and 3D cell cultures, 2D and 3D-spheroid-derived EVs, EVs from DMSO-treated cells, EVs from TMZ-treated cells, GBM genetic markers, and methyltransferases)—to RNA-seq data from normal brain tissue (n = 5) and glioblastoma primary tumors (n = 156) available in The Cancer Genome Atlas (TCGA) (Fig. 7*A*). It is important to note that TCGA provides transcriptomic (RNA-seq) profiling data rather than direct measurements of protein abundance. Therefore, these analyses were used to assess whether genes corresponding to proteins identified in our proteomic datasets are transcriptionally dysregulated in GBM, rather than to directly infer protein-level expression. The protein expression levels of fifty-four genes were found to be increased in GBM primary tumors (indicated by green upward arrows), while fourteen genes were decreased (indicated by red downward arrows). We further examined these genes to determine whether their protein expression levels correlated with patient survival. Among the 68 differentially expressed genes, eight (RPA3, RPL7A, NCAM1, CYBRD1, DPY19L1, HTATSF1, NOP2, and METTL7B) were identified as potential prognostic markers in GBM samples. High protein expression of four genes—RPA3 (*p* = 0.000038), CYBRD1 (*p* = 0.00020), DPY19L1 (*p* = 0.00036), and METTL7B (*p* = 0.00066)—was associated with an unfavorable prognosis in GBM. In contrast, high expression of RPL7A (*p* = 0.000093), NCAM1 (*p* = 0.00015), HTATSF1 (*p* = 0.00040), and NOP2 (*p* = 0.00052) was associated with a more favorable prognosis (Fig. 7*B*).

## Discussion

In this study, we investigated the effect of TMZ treatment on the protein profiles of EVs derived from the GBM U87 cell line grown under 2D and 3D-spheroid culture conditions. Notably, the difference in EV release between 2D and 3D cultures was substantial, with 3D-spheroids secreting more EVs than their 2D counterparts. This observation aligns with previous findings that suggest 3D tumor cell culture models more accurately mimic the *in vivo* microenvironment (32, 33), where enhanced EV secretion may be linked to increased cell-cell interaction (34, 35), hypoxia (36), or stress responses (37). In contrast, TMZ treatment stimulated the release of larger EVs in 2D cultures, likely because more efficient drug diffusion in monolayers than in 3D-spheroids promotes plasma-membrane budding of larger vesicles (Fig. 2). While 3D-spheroids exhibited reduced apoptotic signatures and attenuated proteomic remodeling following TMZ exposure compared to 2D cultures, these findings are interpreted as indicative of reduced sensitivity rather than formal TMZ resistance, which would require dedicated dose–response or longitudinal viability analyses.

In addition to revealing significant differences in EV quantity and size between 2D and 3D models, the multiplex bead-based flow cytometry analysis revealed the heterogeneity and tumor-specific molecular signature of GBM-derived EVs (38, 39) (Fig. 3). In GBM, CD44 is widely recognized as a key cancer stem cell (CSC) marker, and when co-expressed with nestin, it predicts a poor prognosis (40). Its overexpression correlates with tumor aggressiveness (41), mesenchymal subtype enrichment (42), and resistance to therapy, particularly following genotoxic stress from TMZ or radiation (43). The co-expression of CD44, CD29 (β1 integrin), and CD49e (α5 integrin) on EVs derived from both 2D and 3D GBM U87 cultures points to a coordinated adhesion and signaling network governing tumor invasiveness and plasticity. In the GBM microenvironment, these molecules frequently collaborate to mediate ECM adhesion, particularly to fibronectin and hyaluronic acid, which are abundant in the tumor stroma (44). Importantly, CD44 can act as a co-receptor with integrins, including α5β1, potentiating the activation of downstream oncogenic cascades such as MAPK and Wnt/β-catenin (45, 46). This molecular interplay reinforces GBM cell adaptation to hostile environments and supports epithelial-to-mesenchymal transition (EMT), a hallmark of therapy-resistant GBM subtypes (47). The detection of this CD44–integrin module in EVs underscores the hypothesis that GBM-derived vesicles are not passive byproducts but rather selectively packaged nanocarriers with functional adhesion and signaling proteins able to modulate recipient cell phenotypes (48). Therapeutically, targeting α5β1 integrin with liposomes enabled the delivery of a small-molecule inhibitor of the JAK/STAT pathway and STAT3 siRNA directly to GBM cells in the brain. This approach effectively crossed the blood–brain barrier and significantly suppressed tumor growth in mice, highlighting its potential as a promising treatment strategy for GBM (49).

Western blot analysis also identified β-catenin in all samples, with mobility shifts observed in 2D cell cultures treated with increasing concentrations of TMZ. While these mobility shifts are consistent with post-translational modifications, such as phosphorylation or ubiquitination, definitive assignment of specific modifications would require targeted validation approaches, such as band excision followed by MS-based PTM analysis (50). β-catenin serves as a convergence point for CD44 and integrin signaling, integrating inputs from ECM adhesion, hyaluronan engagement, and Wnt ligand activation. In the EV context, the presence of CD44, CD29, and CD49e suggests that these vesicles may carry functional components of this signaling network to nearby cells, potentially influencing β-catenin activity in recipient populations. The observed electrophoretic shifts in β-catenin under TMZ exposure indicate altered intracellular signaling and may correlate with selective packaging of modified proteins or regulatory RNAs into EVs. This observation adds a layer of complexity to the role of EVs in GBM, positioning them as vehicles for the propagation of oncogenic signals within the tumor niche. Collectively, our findings suggest that the CD44–integrin–β-catenin axis is preserved in GBM-derived EVs and may serve as both a biomarker of tumor aggressiveness and a functional mediator of intercellular communication. Targeting this axis could offer novel strategies for impairing tumor–stroma crosstalk, inhibiting EV-mediated signaling, and overcoming resistance to conventional therapies.

Our proteomic analysis of U87 GBM cells and their secreted EVs under 2D and 3D culture conditions revealed treatment-dependent changes in protein composition (24) (Fig. 4). In cell lysates, principal component analysis (PCA) revealed dose-dependent clustering for 2D cell cultures, whereas 3D-spheroids exhibited minimal separation under increasing TMZ doses, suggesting that 3D cell cultures either buffer TMZ-induced stress more effectively or undergo more subtle proteomic adjustments. Notably, several stress- and survival-related proteins were differentially expressed exclusively in 2D cultures treated with TMZ, including FAK1, FABP5, and SPTLC1. These proteins are associated with lipid metabolism (24), membrane remodeling (51), and focal adhesion signaling—processes crucial for cell survival under genotoxic stress (52). FAK1 is activated by α5β1 integrin engagement, which enhances β-catenin activity by stabilizing it in the cytoplasm and promoting its translocation to the nucleus, where it activates the transcription of Wnt target genes (c-Myc, cyclin D1, AXIN2) (53); mechanistically, this may occur through FAK1-mediated inhibition of GSK-3β, either directly or via downstream PI3K/Akt signaling, thereby preventing β-catenin degradation (54).

In contrast, 3D-spheroids uniquely increased the abundance of proteins, such as SOD1 and LPCAT1, which are linked to redox homeostasis (55) and phospholipid remodeling (56), respectively. The presence of TDP1 and SREK1 in 3D cultures also highlights an adaptive cellular response centered on DNA repair (57) and RNA processing—mechanisms consistent with stem-like, therapy-resistant phenotypes (58). Notably, ZYX and EFNMT were elevated in both 2D and 3D cell culture models under TMZ treatment, suggesting shared responses involving cytoskeletal and translational regulation. ZYX in EVs may promote migration and adhesion changes (59), while EFNMT is a methyltransferase that modifies eukaryotic elongation factor 1A (eEF1A), a multifunctional protein involved in both translation and cytoskeletal regulation. Through post-translational methylation, EFNMT fine-tunes eEF1A’s roles in protein synthesis, actin bundling, and cellular stress responses (60).

Among the TMZ-induced EV proteins from 2D cultures, several were linked to DNA repair (XRCC6 (61), MAPK1 (62), EIF3CL), ribosome biogenesis (RPL7A, RPL10A, RPL19) (63), and proteostasis (PA2G4 (64), ADRM1 (65)), suggesting active packaging of proteins involved in maintaining genomic stability and translational capacity (Fig. 5). Notably, histone H1.10 was also enriched in EVs following high-dose TMZ, possibly reflecting chromatin remodeling events triggered by DNA damage (66). In contrast, four proteins were differentially expressed in 3D-spheroid-derived EVs in response to TMZ 200 μM. Interestingly, the abundance levels of ARG1 and SERPINB12 were increased, suggesting potential modulation of the immune microenvironment via arginine metabolism and protease inhibition, respectively. ARG1 catalyzes the conversion of L-arginine into L-ornithine and urea, a critical step in the urea cycle (67). In the GBM microenvironment, ARG1 depletes L-arginine, leading to suppressed proliferation of T-cells and NK cells, thus facilitating immune evasion (68). Moreover, GBM-derived EVs are enriched in ARG1 and can reprogram microglia in the tumor microenvironment by downregulating genes involved in tumor sensing and killing, while upregulating genes that promote tumor spread (69). The downregulation of RPL15 and MYO1C may indicate selective suppression of ribosomal and cytoskeletal components, further supporting the stress-resistant, quiescent-like phenotype of 3D-spheroids.

A key observation was the partial overlap in TMZ-responsive proteins between cell lysates and their corresponding EVs. Proteins such as XRCC6, MAPK1, and FABP5, differentially expressed in 2D cell cultures, were also enriched in their EV counterparts, supporting the idea that EV cargo can reflect the intracellular signaling status. This coordinated EV packaging may contribute to the horizontal transfer of stress-adaptive signals, potentially priming recipient cells to enhance DNA repair, resist apoptosis, or escape immune recognition. Moreover, the selective inclusion of translation machinery components (e.g., RPL10A, EIF3CL) and signaling mediators (e.g., MAPK1, PA2G4) into EVs upon TMZ exposure suggests that GBM EVs not only reflect stress-induced proteomic remodeling but may actively propagate therapy resistance. These findings align with the concept that GBM-derived EVs contribute to intratumoral heterogeneity and treatment evasion by transferring oncogenic and pro-survival cues to neighboring or distant cells (70).

TMZ, the frontline chemotherapeutic agent for GBM, exerts its cytotoxic effect primarily through methylation of the O6 position of guanine residues in DNA. This modification triggers mismatch repair-dependent DNA damage, replication fork collapse, and ultimately apoptosis (71). However, the emergence of resistance—often driven by adaptive epigenetic rewiring—remains a major obstacle to durable clinical responses (8). We investigated 25 methyltransferases spanning DNA (DNMT1, DNMT3A), RNA (METTL3, METTL7B), and protein (PRMT1, PRMT5) targets, alongside metabolic methyl donors such as SHMT2 (Fig. 6). In both 2D and 3D-spheroid U87 GBM cells, we observed a TMZ dose-dependent downregulation of methyltransferases, which is consistent with impaired epigenetic regulation and biosynthetic stress following DNA damage. These findings are further reinforced by previous studies demonstrating that DNMT3B is upregulated in TMZ-resistant GBM cells, such as U251-TMZ, and that its silencing via siRNA enhances chemosensitivity to TMZ by promoting apoptosis and reducing cell proliferation (72). Similarly, RNA methyltransferases such as METTL7B—associated with the m^6^A epitranscriptomic mark—were significantly reduced in cellular compartments following TMZ exposure. These findings align with a shift toward global hypomethylation and reduced transcriptional fidelity, characteristic of GBM under genotoxic pressure (73).

Paradoxically, several methyltransferases were enriched in EVs released by TMZ-treated cells, suggesting a non-random, regulated export of epigenetic regulators. In 2D-derived EVs, we observed increased levels of FBL, a key nucleolar rRNA methyltransferase involved in ribosome biogenesis and translational control (74); SHMT2, a serine-glycine metabolism enzyme linked to one-carbon methyl donor pools and oxidative stress adaptation (75); and TRM5, a mitochondrial tRNA methyltransferase that ensures translational fidelity under metabolic stress (76). These proteins may contribute to EV-mediated reprogramming of recipient cells in the tumor microenvironment (TME), enhancing stromal plasticity or promoting GBM stem-like cell (GSC) maintenance. FBL and SHMT2 were also elevated in 3D-derived EVs under TMZ, reinforcing their conserved role in vesicle-based adaptation. Importantly, SHMT2 has recently emerged as a metabolic node linking serine metabolism to redox balance and epigenetic state, supporting cell survival under hypoxia and DNA damage (77)—hallmarks of the GBM TME. Its increased abundance in EVs could enhance pro-tumorigenic adaptation in neighboring cells or even act as a metabolic “decoy” to buffer oxidative stress (78). Similarly, the presence of TRM5 in EVs may reflect a strategy to safeguard mitochondrial translation in a subset of cells, supporting clonal selection of resistant populations. Enrichment of methylation-related enzymes (FBL, TRM5, SHMT2) in EVs from 2D cultures may reflect stress or metabolic adaptation to TMZ. However, these data do not demonstrate active sorting and may instead result from altered protein turnover, passive incorporation during vesicle biogenesis, or stress-induced redistribution. Therefore, these EV signatures should be interpreted as indicators of altered cellular states rather than direct evidence of targeted epigenetic signaling.

Analysis of The Cancer Genome Atlas (TCGA) (79) data revealed EV-associated proteins linked to either poor or favorable prognosis in GBM (Fig. 7). Among the poor-prognosis group, RPA3—a single-strand DNA-binding protein crucial for replication fork stability and homologous recombination repair—was increased in TMZ-treated U87 cells and EVs, consistent with heightened replication stress and genomic instability, features associated with poor response to alkylating agents (80); CYBRD1, an iron metabolism protein mediating ferric reduction, was also elevated; its role in oxidative stress modulation, ferroptosis resistance, and metabolic plasticity may contribute to therapy resistance (81); DPY19L1, a C-mannosyltransferase involved in protein glycosylation and Golgi function, though understudied in GBM, may promote tumor progression via enhanced protein trafficking, secretory activity, or glyco-editing of surface proteins linked to adhesion and immune evasion (82); METTL7B, a lipid metabolism–associated methyltransferase, was decreased intracellularly but selectively retained in EVs, suggesting active sorting as a means to influence the tumor microenvironment or modulate RNA methylation pathways, with its elevation correlating with poor survival (83). In contrast, favorable-prognosis proteins included RPL7A, a ribosomal large subunit component linked to translational robustness (84), increased in EVs from TMZ-treated cells; NCAM1, a neural differentiation marker potentially maintaining neuroglial identity and counteracting mesenchymal transformation (85); HTATSF1, a splicing and transcription elongation regulator that, despite its oncogenic associations, may enhance splicing fidelity or immune surveillance in certain GBM contexts (86); and NOP2, an rRNA methyltransferase involved in rRNA modification and ribosome biogenesis (87) typically considered pro-oncogenic (88). Elevated in TMZ-treated EVs, NOP2 might be linked to controlled proliferation, classical differentiation states, and improved treatment response. Importantly, mRNA–protein concordance in cancer is often limited due to post-transcriptional regulation, protein turnover, and context-dependent secretion mechanisms, such as EV-mediated cargo sorting; therefore, TCGA transcriptomic data should be viewed as complementary to, rather than a surrogate for, the proteomic alterations identified in this study.

From a clinical perspective, these findings highlight the potential of EV proteomics to address key challenges in GBM management. Given the limited accessibility of tumor tissue and the delay between biological progression and radiographic detection, EVs represent a promising non-invasive approach to monitor treatment response and resistance. The enrichment of metabolic, immune-modulatory, and epigenetic regulators in EV cargo following TMZ exposure supports their relevance as dynamic biomarkers. As a liquid biopsy strategy, EV profiling from blood or CSF could complement current surveillance methods and enable longitudinal monitoring of tumor evolution. Although these observations remain preclinical, they underscore the potential of EV proteomics to inform patient stratification and therapeutic decision-making.

We acknowledge that the use of a single, patient-derived glioblastoma cell line represents a limitation of this study. While cultured U87 cells provide a controlled and reproducible system, they do not fully capture the complexity and heterogeneity of primary GBM tumors. Therefore, this work should be viewed as a discovery framework, and validation in patient-derived multi-cell type heterogeneous models and clinical EV samples will be essential to confirm translational relevance. Future studies integrating publicly available patient-derived GBM proteomic datasets will also be valuable for determining whether the TMZ-associated cellular and EV signatures identified here are recapitulated in clinical specimens.

In conclusion, our integrated analysis of cellular and EV proteomes across 2D and 3D GBM models demonstrates that TMZ induces cell culture architecture-dependent stress-adaptive responses. While 2D cultures exhibit dynamic proteomic remodeling, 3D-spheroids display a more stable phenotype consistent with therapy resistance. EVs not only reflect these differences but also selectively package proteins involved in genomic stability, metabolism, and epigenetic regulation, suggesting a role in intercellular communication and tumor adaptation. The integration of these findings with TCGA data further supports the clinical relevance of candidate EV-associated proteins as potential biomarkers and therapeutic targets. Collectively, our results underscore the importance of 3D modeling in GBM research and highlight EV proteomics as a promising approach to study tumor adaptation and resistance.

## Ethics Statement

This study was conducted in accordance with the ethical standards and best practice guidelines outlined by the Committee on Publication Ethics (COPE) and the International Committee of Medical Journal Editors (ICMJE). The research involved only commercially available human GBM U87 cell lines and did not include the use of human participants, patient data, or animal subjects. Therefore, no institutional ethics approval or informed consent was required. All experimental procedures were performed in compliance with institutional biosafety regulations.

## Data Availability

All raw proteomic data generated in this study have been deposited in the ProteomeXchange Consortium via the PRIDE database under the project accession number PXD069801. The repository includes the raw mass spectrometry files, processed proteomic datasets, and the Proteome Discoverer output files (.pdResults) containing the annotated MS/MS spectra used for protein identification and downstream data analysis. A detailed description of the experimental datasets, including proteomic data spreadsheets and other supplementary materials, is provided in the Supplemental Data section.

## Supplemental data

This article contains supplemental data.

## Conflict of interest

The authors declare that they have no conflicts of interest with the contents of this article.
