# Supplementary material for: Extracellular Vesicles From Glioblastoma Cells Reflect 2D vs. 3D Culture Adaptation and Resistance to Temozolomide
Source: Mol Cell Proteomics. 2026 Jun 24;25(7):101610. doi: 10.1016/j.mcpro.2026.101610 (PMC13393710; doi:10.1016/j.mcpro.2026.101610)
Supplement: Supplementary Figures [file mmc5.docx]

**Supplementary information**

**Extracellular Vesicles from Glioblastoma Cells Reflect 2D vs. 3D Culture Adaptation and Resistance to Temozolomide**

Getulio Pereira de Oliveira Junior^1,2*^, Alan Zimmerman^1^, Cintia C Palu^3^, Vivian Tran^4^, Gabrielle Bogut^5^, Stephanie Chidester^6^, John Tigges^7^, Rafael A. Vega^8^, Elena Aikawa^5^, Jennifer Jones^6^, Ionita C. Ghiran^4^, and Alexander R. Ivanov^1*^

1. Department of Chemistry and Chemical Biology, Barnett Institute of Chemical & Biological Analysis, Northeastern University, Boston, MA, USA;
2. Graduate Program of Genomic Sciences and Biotechnology, Catholic University of Brasilia, Brasilia, Brazil;
3. Expert Biomarker Data Scientist, Sanofi, Cambridge, UK;
4. Department of Anesthesia, Beth Israel Deaconess Medical Center, Harvard Medical School, Boston, MA, USA;
5. Center for Interdisciplinary Cardiovascular Sciences, Brigham and Women’s Hospital, Harvard Medical School, Boston, MA, USA;
6. Laboratory of Pathology, Center for Cancer Research, National Cancer Institute, Bethesda, MD, USA;
7. Nanoflow Cytometry Core Facility, Beth Israel Deaconess Medical Center, Harvard Medical School, Boston, MA, USA;
8. Division of Neurosurgery, Beth Israel Deaconess Medical Center, Harvard Medical School, Boston, MA, USA

* Corresponding authors: [getulio.junior@a.ucb.br](mailto:getulio.junior@a.ucb.br) and a.ivanov@northeastern.edu

Department of Chemistry and Chemical Biology, Barnett Institute for Chemical & Biological Analysis, Northeastern University, 140 Fenway, Boston, MA 02115

**Table of contents**

Supplementary figure 1 Page 3

Supplementary figure 2 Page 4

Supplementary figure 3 Page 5

Supplementary figure 4 Page 6

Supplementary figure 5 Page 8

Supplementary figure 6 Page 9

Supplementary figure 7 Page 11

Supplementary figure 8 Page 12

Supplementary figure 9 Page 14

Supplementary figure 10 Page 15

Information about supplementary tables Page 16


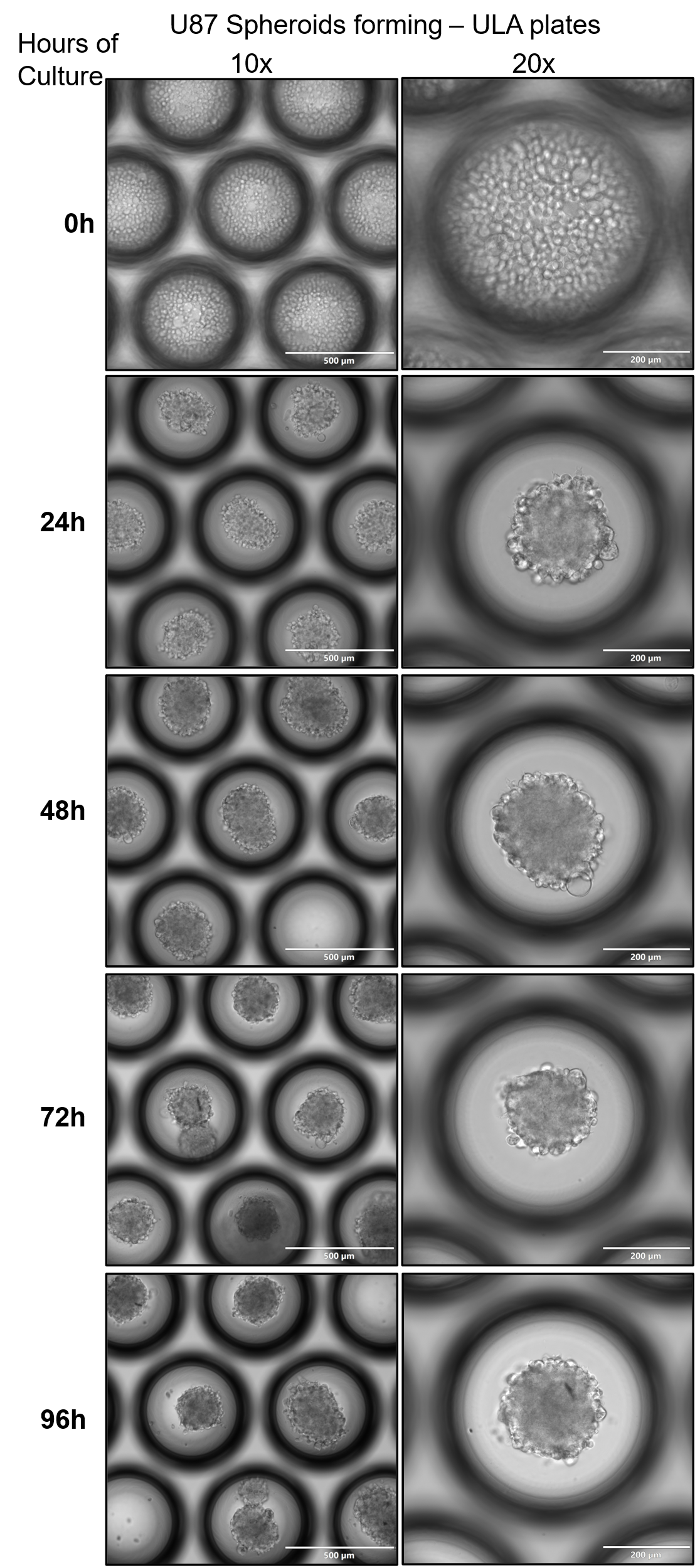


**Supplementary figure 1.** Formation and Growth of U87 Spheroids in Ultra-Low Attachment Plates. Brightfield images depict the formation and development of U87 spheroids over time in ultra-low attachment (ULA) plates. The left panel shows an overview of multiple wells with spheroids forming at different stages, while the right panel provides a close-up view of individual spheroids. The images demonstrate the progression from initial cell aggregation to compact spheroid formation, with the diameter of the spheroids increasing as the cells proliferate and self-assemble. Scale bars: 500 µm (left) and 200 µm (right).


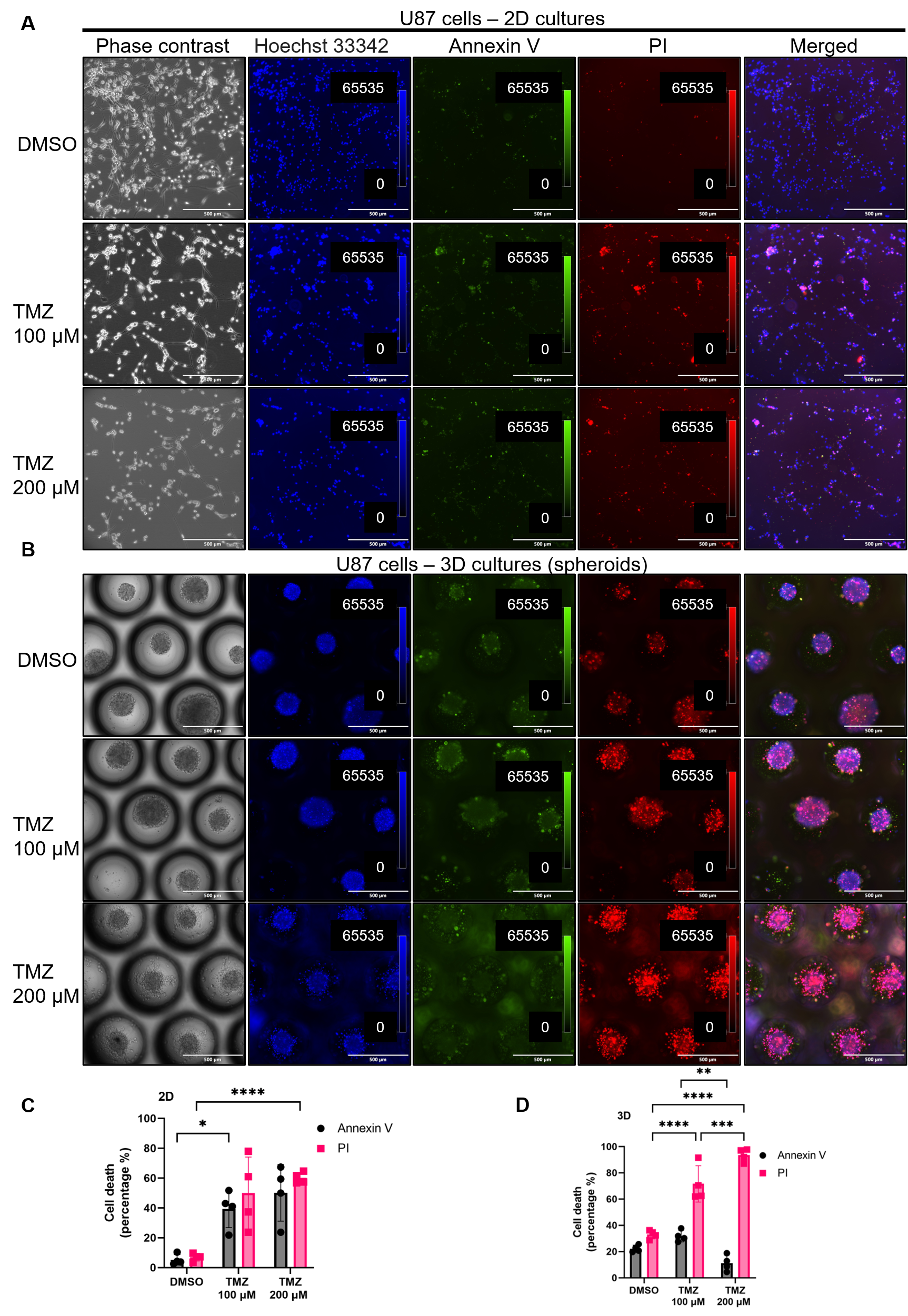


**Supplementary figure 2.** Temozolomide Induces Cell Death in U87 2D and 3D Spheroid Models.

(A) U87 cells cultured under 2D conditions were treated with DMSO (control), 100 µM, or 200 µM TMZ, followed by Annexin V and propidium iodide (PI) staining to assess apoptosis and necrosis. Fluorescence microscopy shows nuclei (Hoechst, blue), early apoptotic cells (Annexin V, green), and late apoptotic/necrotic cells (PI, red). (B) The same staining protocol was applied to 3D spheroids. (C) In 2D cultures, quantification of apoptotic and necrotic cells (normalized to total nuclei) revealed a significant, dose-dependent increase in both forms of cell death (p<0.05 for apoptosis at 100 µM; p<0.0001 for necrosis at 200 µM, Two-way ANOVA with Šídák’s test). (D) In 3D cultures, TMZ induced a modest increase in apoptosis but a marked, dose-dependent rise in necrosis, significant at both 100 µM and 200 µM (p<0.0001).


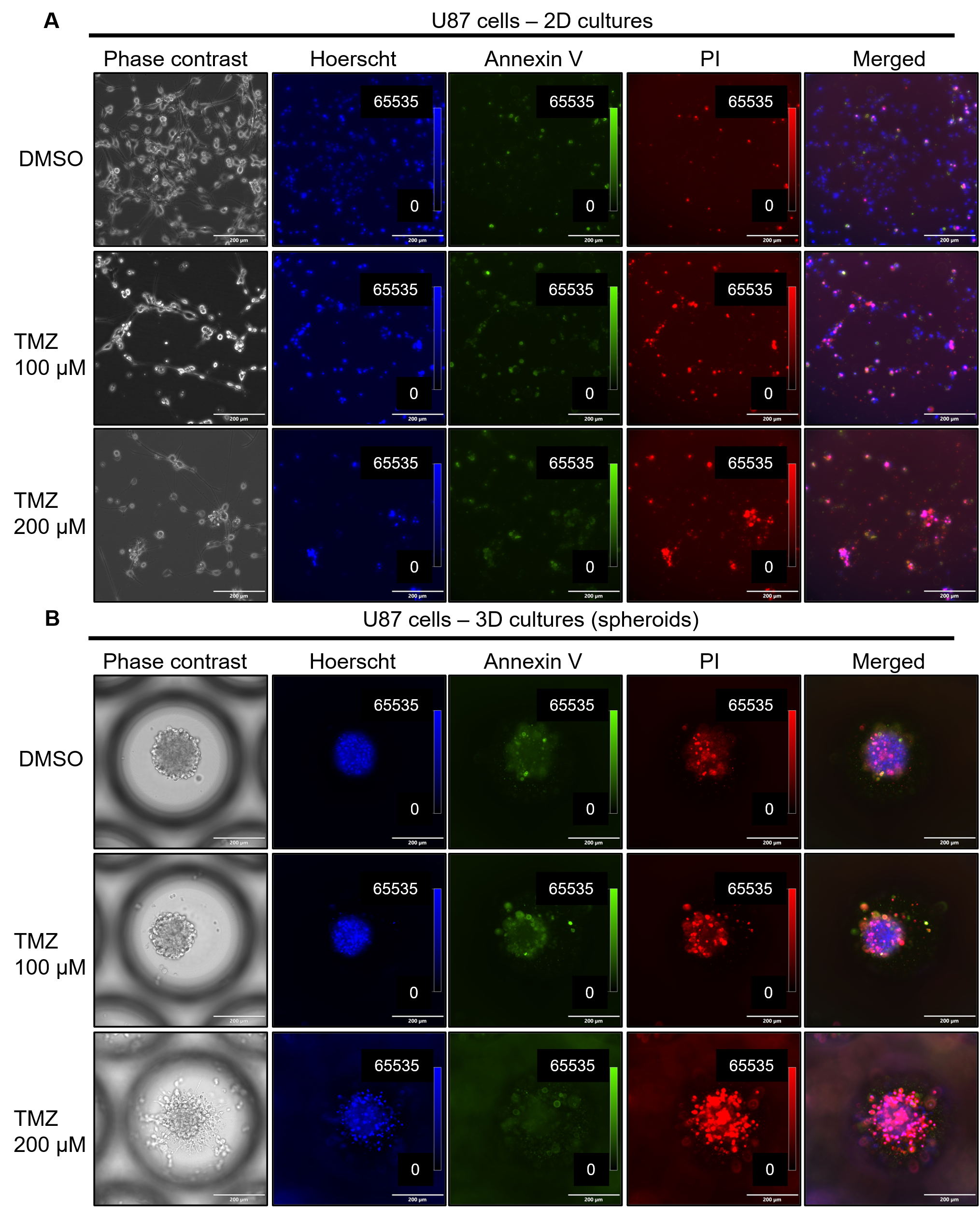


**Supplementary figure 3.** Fluorescence Microscopy of U87 Cells and Spheroids Treated with TMZ. Fluorescence microscopy images show U87 cells cultured under 2D (top panels) and 3D spheroid conditions (bottom panels) stained with Hoechst 33342 (blue, nucleus), Annexin V (green, apoptotic cells), and Propidium Iodide (red, necrotic cells). In the 2D monolayer (top), increasing apoptosis and necrosis levels are visible following treatment with temozolomide (TMZ). In the 3D spheroids (bottom), a similar pattern is observed, with fluorescence indicating the localization of apoptosis and necrosis within the spheroids. The overlay images (far right panels) combine all channels, providing a comprehensive view of the spatial distribution of apoptotic and necrotic cells in both 2D and 3D cultures. Scale bars: 50 µm.


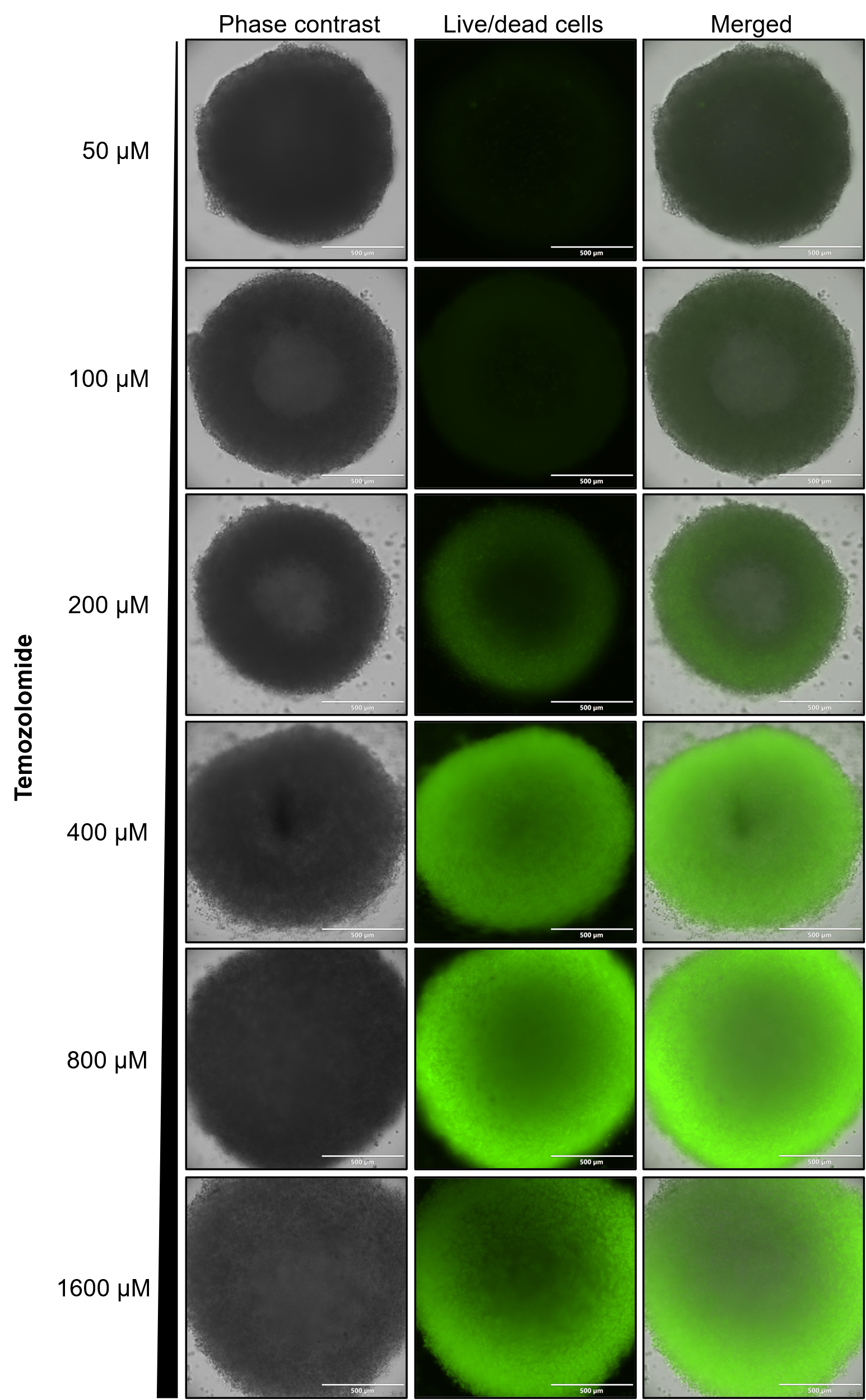


**Supplementary figure 4.** Fluorescence and Brightfield Imaging of U87 Spheroids Treated with Increasing Concentrations of Temozolomide. Brightfield (left) and corresponding fluorescence (center and right) images depict U87 spheroids treated with increasing concentrations of temozolomide (TMZ), ranging from 50 µM to 1600 µM. The spheroids were generated using ~20,000 U87 cells per well, resulting in larger structures exceeding 1 mm in diameter. The experiment was performed using a live/dead cell staining kit, where fluorescent cells indicate dead cells. As the concentration of TMZ increases, fluorescence intensity becomes more prominent, revealing a higher proportion of dead cells in response to the drug. The brightfield images show the structural integrity of the spheroids, while the fluorescence images highlight the spatial distribution of dead cells, illustrating a dose-dependent cytotoxic effect. Scale bars: 500 µm.


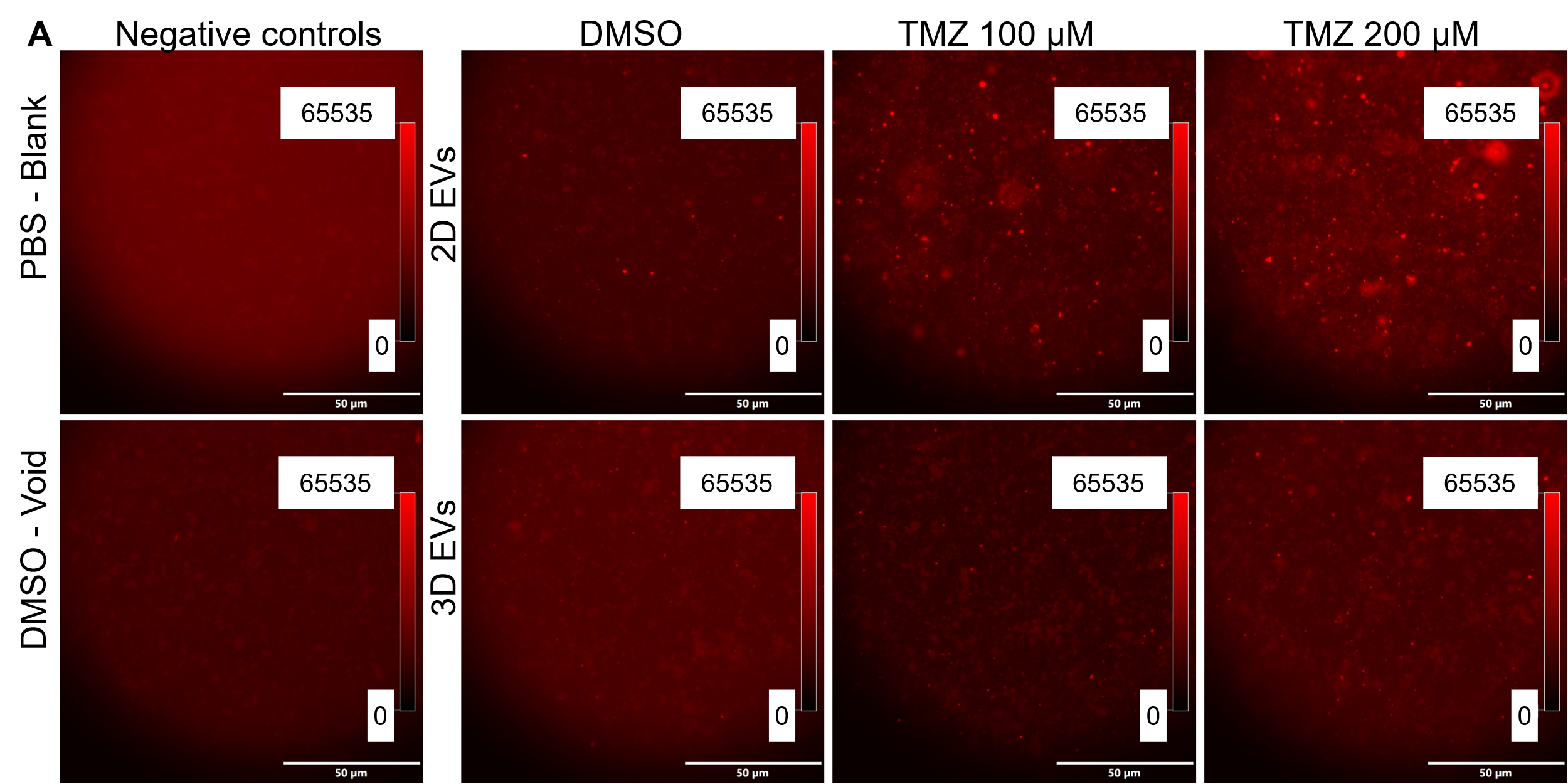


**Supplementary figure 5.** (A) Extracellular vesicles (EVs) isolated from U87 cells cultured under 2D and 3D conditions were labeled with CellMask Red and visualized using fluorescence microscopy. The images reveal EV populations under different culture conditions.


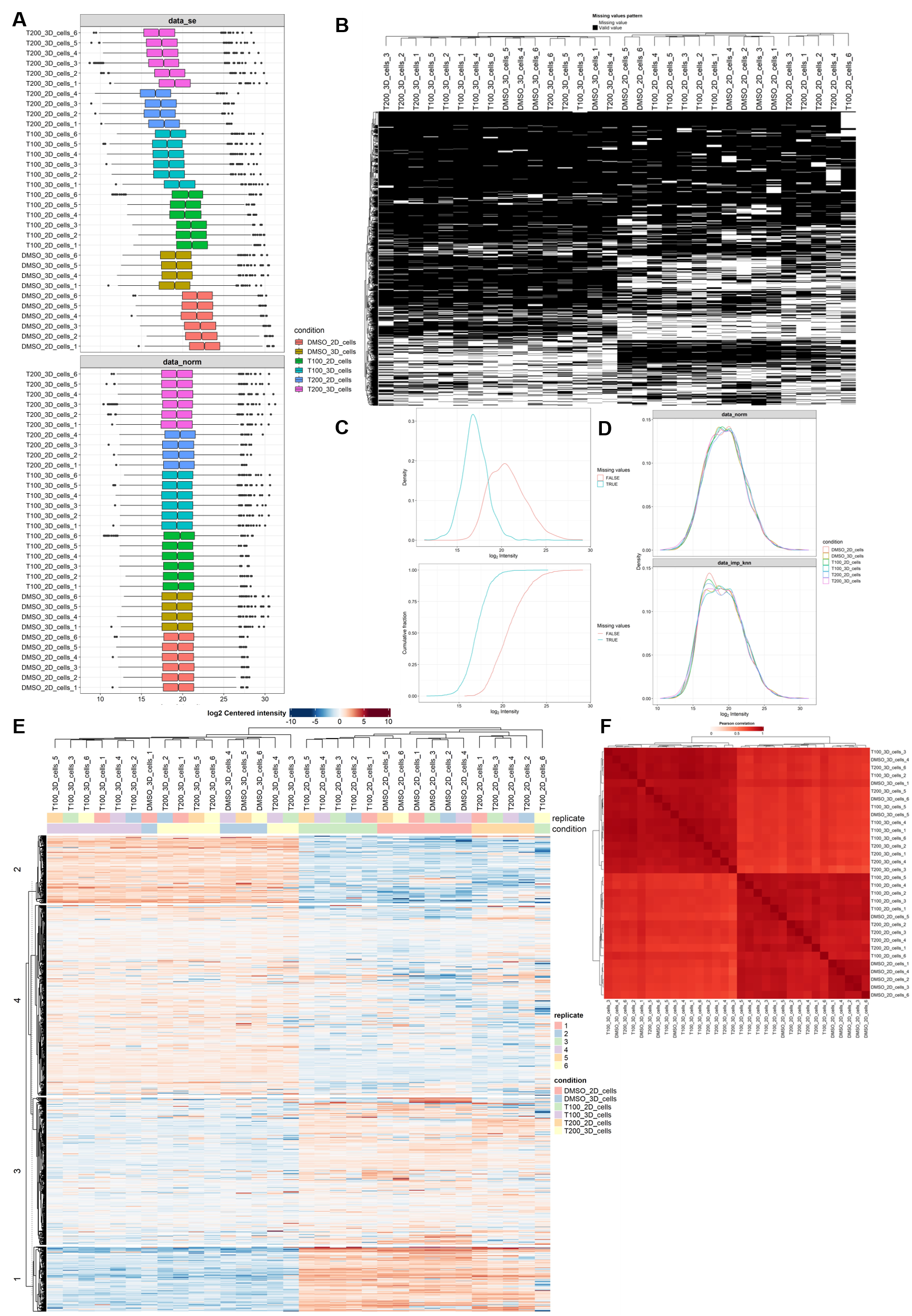


**Supplementary figure 6.** Comparative Proteomic Analysis of U87 Cells Treated with Temozolomide (TMZ) in 2D and 3D Cultures. (A) Boxplots illustrating the distribution of protein intensity values for each sample, highlighting the variability across conditions, including DMSO-treated and TMZ-treated U87 cells in both 2D and 3D cultures. (B) Missing value heatmap indicating the presence of missing proteomic data across the samples, showing areas of incomplete data collection. (C) Density plots representing the distribution of protein intensity values for samples treated with DMSO and TMZ at different concentrations, indicating shifts in protein expression. (D) Empirical cumulative distribution functions (ECDFs) and density distributions for the data sets, illustrating the difference in data completeness and intensity distribution for TMZ-treated and untreated samples. (E) Heatmap of protein expression profiles, showing the differential proteomic expression between U87 cells treated with DMSO, TMZ 100 µM, and TMZ 200 µM, in 2D and 3D cultures. The hierarchical clustering reveals distinct expression patterns between the treatment groups. (F) Correlation heatmap of replicates, displaying the similarity between replicates within each experimental group based on protein intensity measurements, with darker red indicating higher correlation.


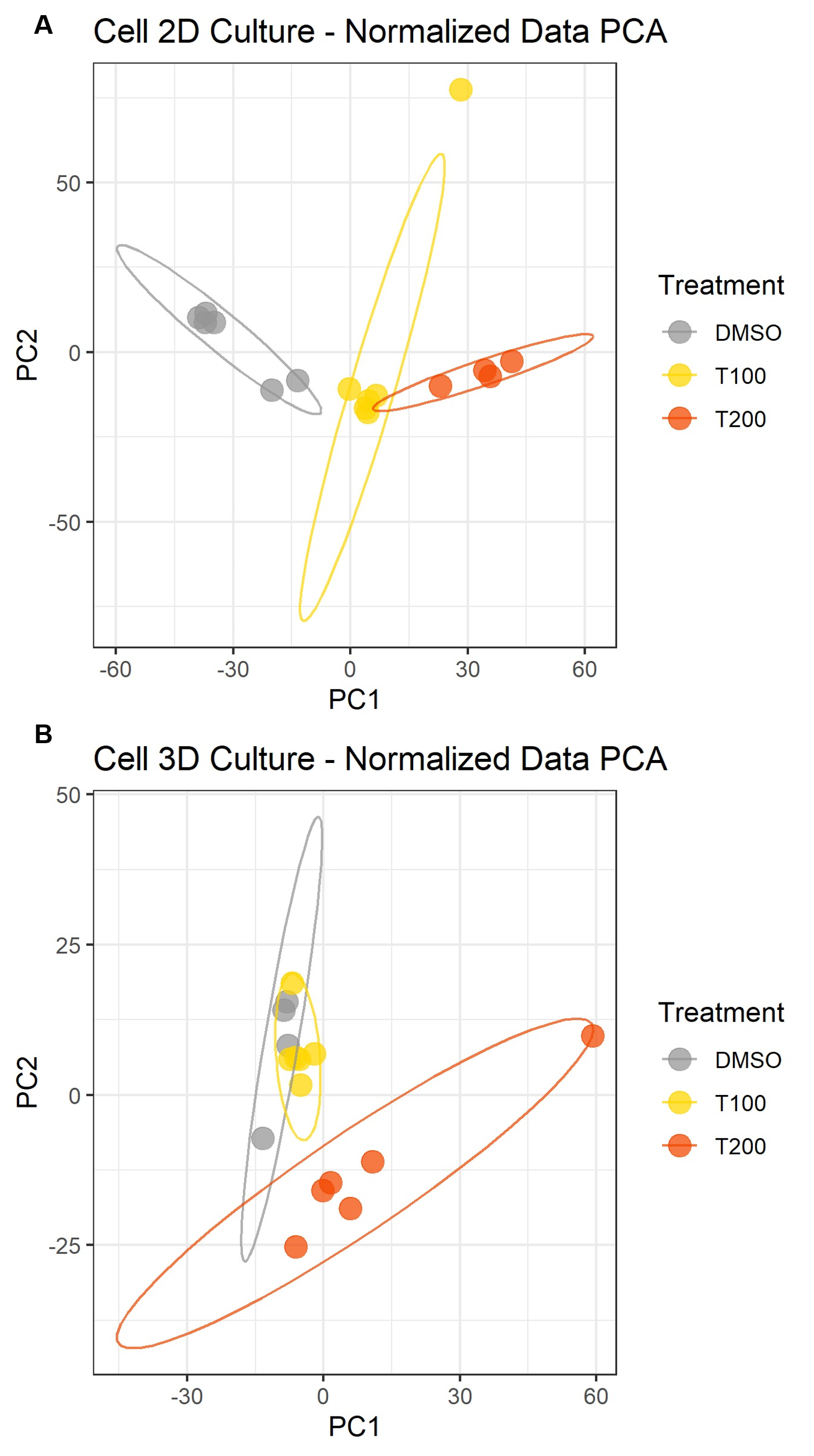


**Supplementary figure 7.** Principal Component Analysis (PCA) of U87 Cells Treated with TMZ in 2D and 3D Cultures. (A) PCA plot of U87 cells cultured in 2D conditions treated with DMSO (gray), TMZ 100 µM (yellow), and TMZ 200 µM (orange). The plot shows a clear separation of the proteomic profiles along PC1 and PC2, with distinct clustering of samples based on treatment groups, indicating TMZ’s dose-dependent effect on the cellular proteome in 2D culture. (B) PCA plot of U87 cells cultured in 3D conditions treated with DMSO (gray), TMZ 100 µM (yellow), and TMZ 200 µM (orange). In 3D cultures, the separation between treatment groups is more pronounced along PC1, with a distinct shift between DMSO and TMZ-treated groups, particularly with 200 µM TMZ, indicating that the proteomic response is more substantial in the 3D culture.


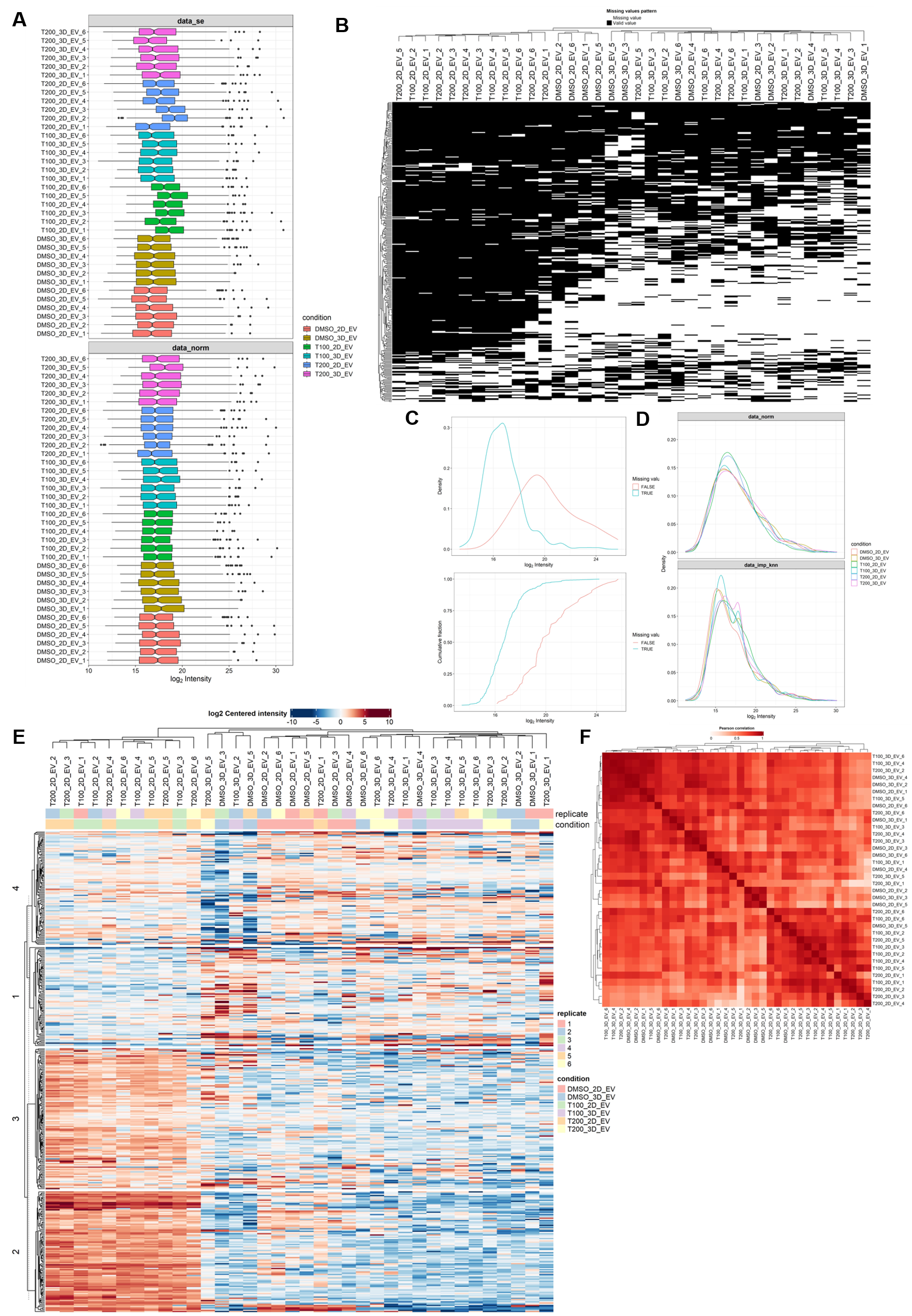


**Supplementary figure 8.** Characterization of EV Proteome from U87 Cells Treated with TMZ in 2D and 3D Cultures. (A) Box plots showing the intensity distribution of identified proteins across all conditions. (B) Missing value heatmap showing the occurrence of missing data across the samples in white. (C) Density and cumulative distribution function plots illustrating the distributions of protein intensities between DMSO and TMZ-treated samples in 2D and 3D cultures. (D) Protein intensity distributions across all conditions in both 2D and 3D EVs. (E) Heatmap showing hierarchical clustering of differentially expressed proteins across the experimental conditions. The clustering reveals distinct patterns in protein expression between 2D and 3D EVs treated with TMZ, with clear separation between treatment groups. (F) Correlation matrix demonstrating the relationship between biological replicates, indicating strong consistency within each experimental group.


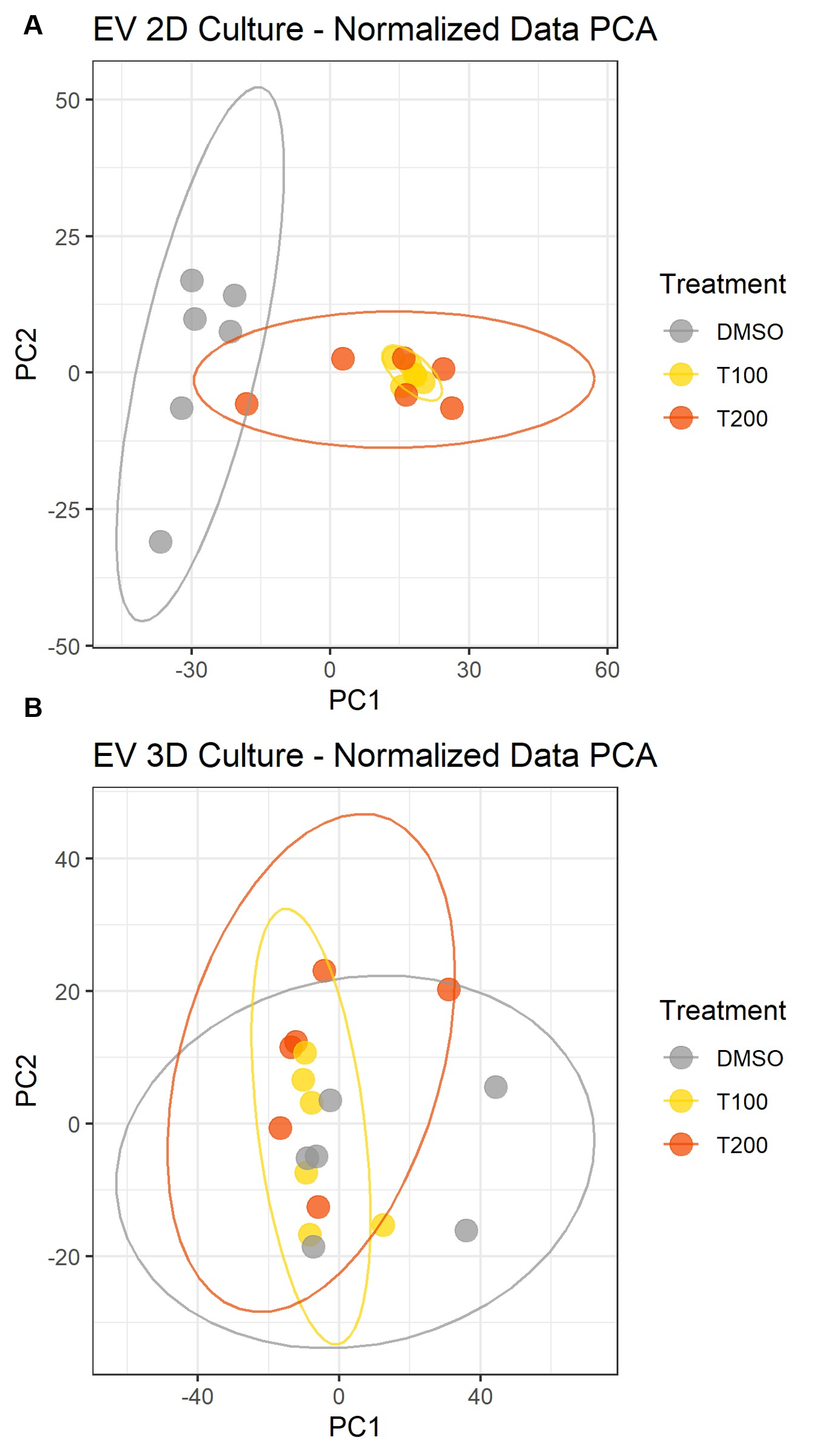


**Supplementary figure 9.** Principal Component Analysis (PCA) of EVs Isolated from U87 Cells Cultured in 2D and 3D Conditions. (A) PCA of EV proteomes from U87 cells cultured in 2D conditions shows separation between DMSO- and TMZ-treated (100 µM and 200 µM) groups along PC1, indicating proteomic differences induced by TMZ treatment. (B) PCA of EV proteomes from U87 cells cultured in 3D conditions reveals a similar separation pattern along PC1 between DMSO and TMZ-treated groups, with a tighter clustering of the TMZ-treated samples. This analysis highlights the proteomic shifts in EVs derived from cells cultured in both 2D and 3D in response to TMZ treatment.


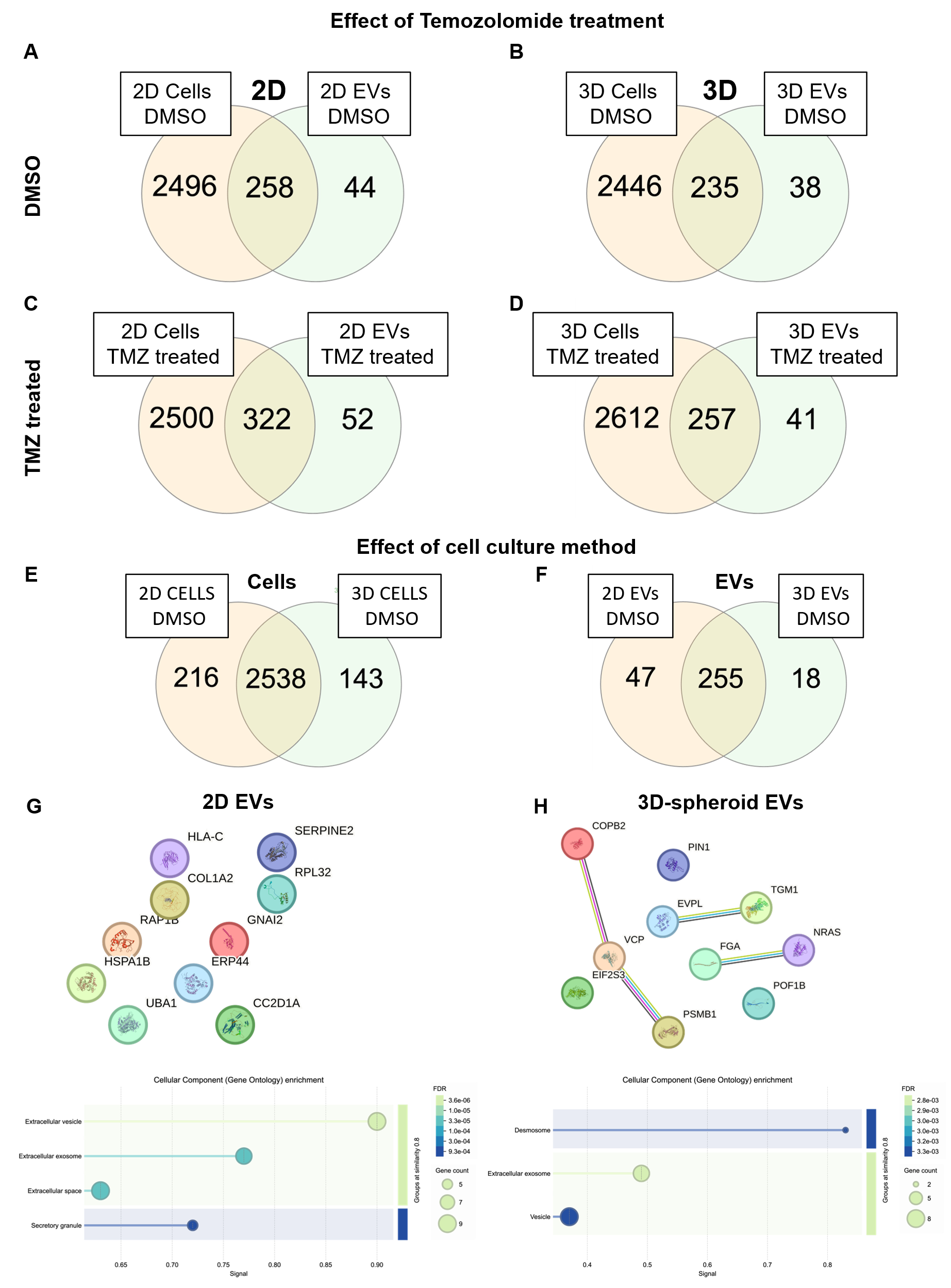


**Supplementary Figure 10.** Venn diagrams and functional analyses of proteins identified in U87 cells and U87-derived extracellular vesicles (EVs) across culture conditions and treatments. (A) Comparison between proteins detected in 2D-cultured U87 cells (DMSO-treated) and their corresponding EVs, highlighting shared and distinct profiles under control conditions. (B) Comparison between proteins detected in 3D-cultured U87 cells (DMSO-treated) and their EVs, showing differences between cells and EVs in a 3D environment. (C) Comparison of proteins identified in 2D-cultured U87 cells treated with TMZ and their EVs, demonstrating chemotherapy-induced changes in both compartments. (D) Comparison of proteins in 3D-cultured U87 cells treated with TMZ and their EVs, illustrating shared and unique proteins in the 3D model after treatment. (E) Venn diagram comparing protein expression between 2D and 3D U87 cells (DMSO-treated), indicating the impact of culture dimensionality on the cellular proteome. (F) Venn diagram comparing proteins in EVs derived from 2D and 3D U87 cells (DMSO-treated), demonstrating dimensionality effects on EV composition. (G) STRING protein–protein interaction network and GO Cellular Component enrichment for the top proteins in 2D-derived EVs, showing enrichment for extracellular vesicle/exosome-related terms and vesicle-mediated transport. (H) STRING network and GO enrichment for the top proteins in 3D-spheroid EVs, highlighting enrichment for the desmosome/cell–cell junction pathway—consistent with the stronger intercellular adhesion characteristic of spheroids.

**Information about supplementary tables:**

**Supplementary Table S1.** Raw and processed label-free quantitative proteomics data for U87 glioblastoma cells cultured under 2D and 3D conditions and treated with DMSO or temozolomide (TMZ). The table includes protein identifiers (UniProt accession and gene name), normalized abundance values for each biological replicate, and group-level summary statistics used for downstream differential expression analysis. Biological replicates correspond to six independent cultures per condition. Data shown represent the output generated by Proteome Discoverer and subsequently processed in R using the DEP package, as described in the Methods. Proteins detected in fewer than 20% of samples were excluded prior to statistical analysis. Normalization and imputation were applied as detailed in the *Proteomics data and bioinformatics analysis* section.

**Supplementary Table S2.** Raw and processed label-free quantitative proteomics data for extracellular vesicles (EVs) isolated from U87 glioblastoma cells cultured under 2D and 3D conditions and treated with DMSO or temozolomide (TMZ). The table includes protein identifiers (UniProt accession and gene name), normalized abundance values for each biological replicate, and group-level summary statistics used for downstream differential expression analysis. EV samples correspond to six independent biological replicates per condition, derived from separately cultured 2D monolayers or 3D-spheroids. Data shown represent the output generated by Proteome Discoverer and subsequently processed in R using the DEP package, as described in the Methods. Proteins detected in fewer than 20% of samples were excluded prior to statistical analysis. Normalization and imputation procedures were applied as detailed in the *Proteomics data and bioinformatics analysis* section.

**Supplementary Table S3.** Differential expression analysis of the cellular proteome of U87 glioblastoma cells cultured under 2D and 3D conditions and treated with DMSO or temozolomide (TMZ). The table reports protein identifiers (UniProt accession and gene name), log2-transformed fold changes between experimental conditions, raw p-values, and multiple-testing–adjusted p-values derived from differential expression analysis performed using the DEP package in R, as described in the Methods. Statistical comparisons were conducted independently for each specified contrast. Only proteins detected in at least 20% of biological replicates were included in the analysis. Six independent biological replicates were analyzed per condition.

**Supplementary Table S4.** Differential expression analysis of the extracellular vesicle (EV) proteome derived from U87 glioblastoma cells cultured under 2D and 3D conditions and treated with DMSO or temozolomide (TMZ). The table reports protein identifiers (UniProt accession and gene name), log2-transformed fold changes for the indicated contrasts, raw p-values, and multiple-testing–adjusted p-values obtained from differential expression analysis performed in R using the DEP package, as described in the Methods. EV and cellular proteomes were analyzed independently. Only proteins detected in at least 20% of biological replicates were included in the analysis. Six independent biological replicates were analyzed per condition.
